# Supplementary material for: A quantitative gibberellin signaling biosensor reveals a role for gibberellins in internode specification at the shoot apical meristem
Source: Nat Commun. 2024 May 8;15:3895. doi: 10.1038/s41467-024-48116-4 (PMC11079023; doi:10.1038/s41467-024-48116-4)
Supplement: Supplementary file 1 — Supplementary Information [file 41467_2024_48116_MOESM1_ESM.pdf]

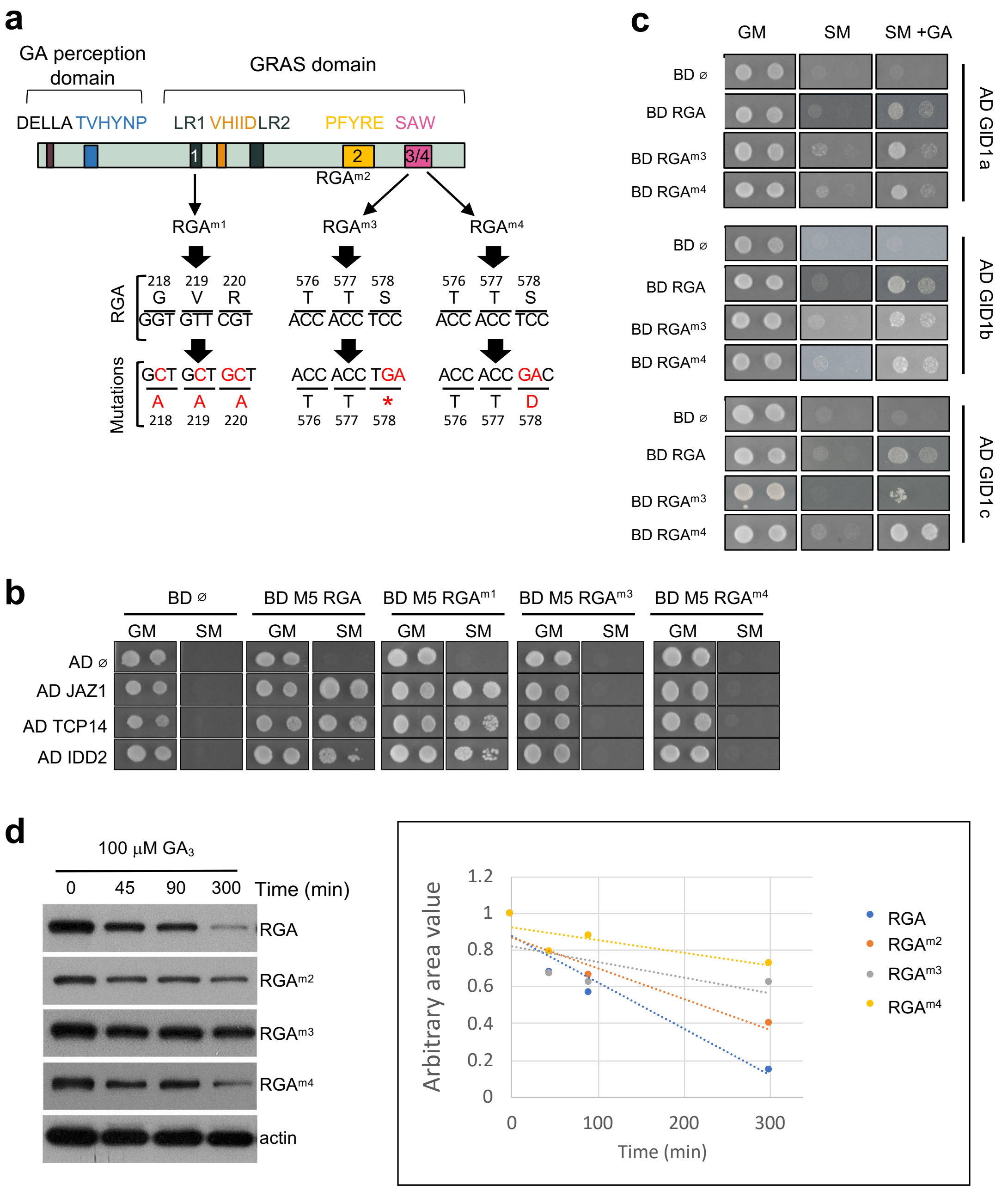

Supplementary Figure 1. Characterization of mutant DELLA proteins.

a, Position of the mutated nucleic acids and amino acids of the four candidate modified DELLA proteins used in the study: RGA^{m1} (G218A, V219A, R220A), RGA^{m2} (H471A, Y472A, Y473A), RGA^{m3} (S578Stop), RGA^{m4} (S578D). *: stop codon. **b**, Pairwise Y2H interaction assays between RGA, RGA^{m1}, RGA^{m3}, RGA^{m4} and three DELLA-interacting proteins, JAZ1, TCP14 and IDD2. Empty pGBKT7 and pGADT7 vectors were included as negative controls. Photos show the growth of the yeast on control media (GM) and on selective media (SM). **c**, Pairwise Y2H interaction assays

between RGA, RGA^{m3}, RGA^{m4} and GID1a, GID1b and GID1c. Photos show the growth of the yeast on control media (GM), selective media (SM) and SM media supplemented with 100 µM GA_3. **d**, Time-course analysis of GA-induced degradation of RGA, RGA^{m2}, RGA^{m3} and RGA^{m4} (same assay and complementary to the one shown in Figure 1d). Left panel: Immunodetection of RGA-GFP, RGA^{m2}-GFP, RGA^{m3}-GFP and RGA^{m4}-GFP proteins respectively in *35S::RGA-GFP*, *35S::RGA^{m2}-GFP*, *35S::RGA^{m3}-GFP* and *35S::RGA^{m4}-GFP N. benthamiana* agro-infiltrated leaves treated with 100 mM cycloheximide (CHX) and 100 µM GA_3 for the indicated times. Actin is used as sample loading control. Right panel: Quantification of the immunoblot signals depicted as a graph. Values indicate RGA-GFP, RGA^{m2}-GFP, RGA^{m3}-GFP and RGA^{m4}-GFP signals relative to actin signals. The experiment was repeated twice with similar results.

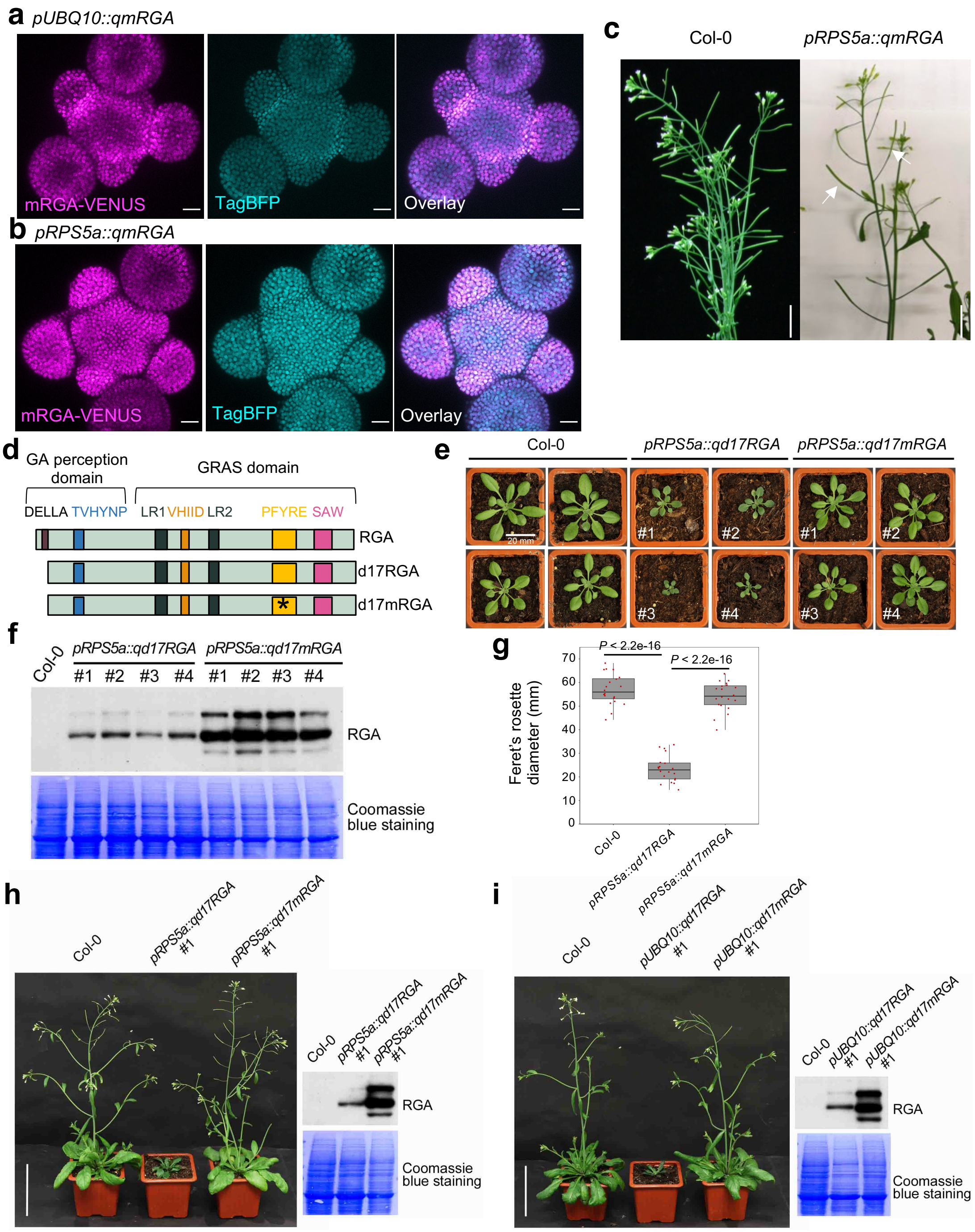

Supplementary Figure 2. qmRGA activity is not interfering with plant growth. **a-b**, Fluorescence signals of mRGA-VENUS, TagBFP-NLS and merge channels in *pUBQ10::qmRGA* (a) and *pRPS5a::qmRGA* (b) inflorescence SAM. The experiment was repeated twice with similar results.**c**, Representative growing wild-type (Col-0) and *pRPS5a::qmRGA* plants with flowers and elongated siliques indicative of normal fertility (arrows). The experiment was repeated twice with similar results. **d**, Schematic representation of d17RGA, a mutant version of RGA, deleted of its N-terminal DELLA

domain involved in the contact with GID1, rendering the protein insensitive to GA. An asterisk indicates the mRGA mutation. **e,f**, Representative 3 weeks old T1 independent transgenic *pRPS5a::qd17RGA* and *pRPS5a::qd17mRGA* plants and Col-0 controls (**e**) and immunodetection of d17RGA-VENUS and d17mRGA-VENUS protein levels using RGA antibodies (**f**). **g**, Boxplot representations of the Feret's rosette diameter of 3 weeks old wild-type (Col-0, n = 20 biological independent plants), *pRPS5a::qd17RGA* (n = 20 biological independent plants) and *pRPS5a::qd17mRGA* (n=20 biological independent plants). Center lines show the medians and box limits indicate the 25th and 75th percentiles. Whiskers indicate minima and maxima as determined by R software. *P* values are determined by R software using two-tailed Welch t-test. **h**,**i**, Representative *pRPS5a::qd17RGA* and *pRPS5a::qd17mRGA* (**h**) and *pUBQ10::qd17RGA* and *pUBQ10::qd17mRGA* (**i**) adult plants (and Col-0 controls). On the right is shown the corresponding immunodetection of d17RGA-VENUS and d17mRGA-VENUS protein levels using RGA antibodies. The experiment was repeated twice with similar results. Scale bars = 20 µm (a, b), 1 cm (c), 20 mm (e), 7 cm (h,i).

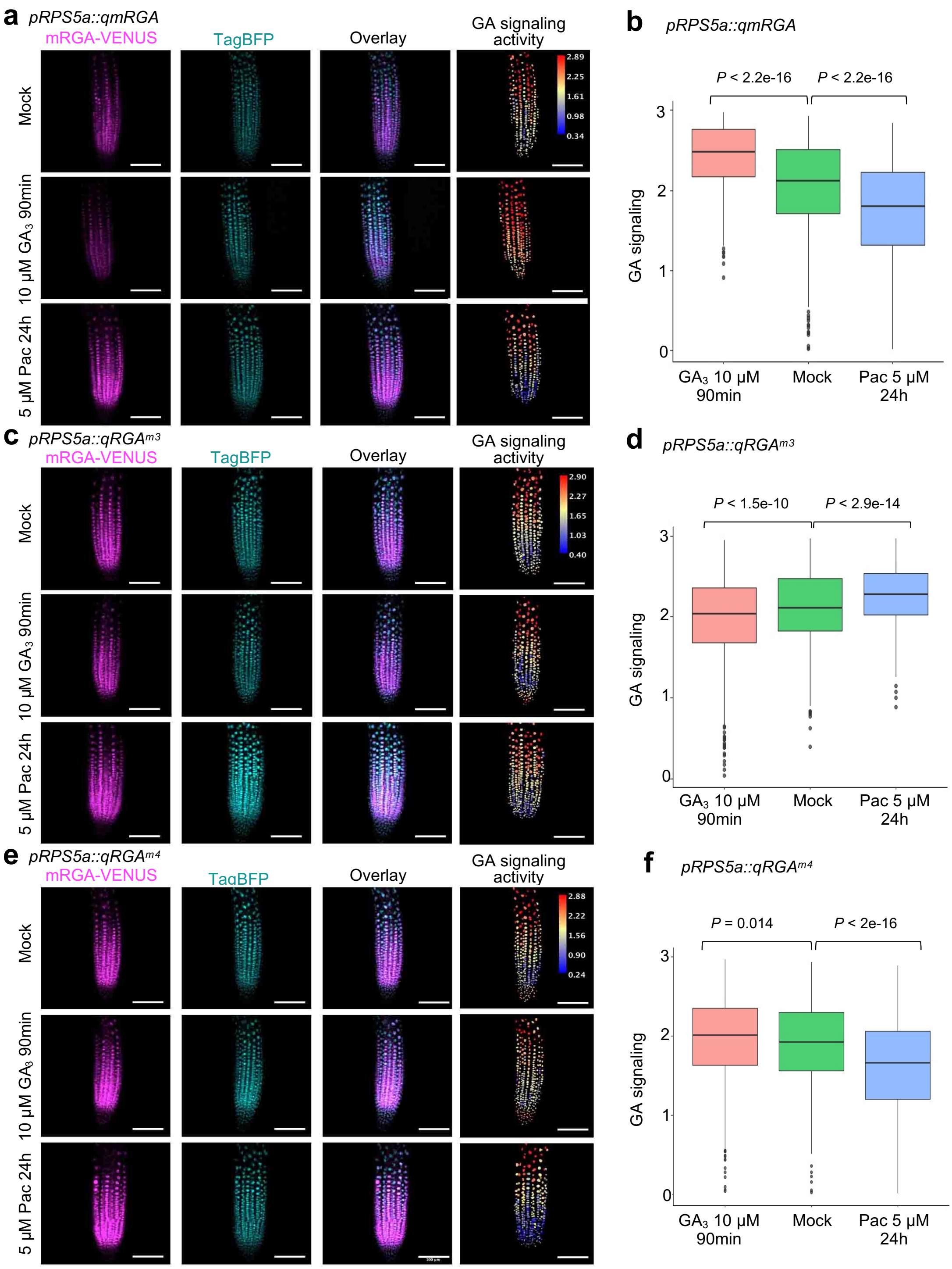

Supplementary Figure 3. Behavior of the different GA signaling sensor lines in response to changes in GA levels in seedling root.

Representative confocal images of VENUS and TagBFP signals, and corresponding heatmap representation of GA signaling activity in seedling root tips of 7-d-old *pRPS5a::qmRGA* (**a**), *pRPS5a::qRGA*^{m3} (**c**) and *pRPS5a::qRGA*^{m4} (**e**) seedlings treated with 10 µM GA_3 for 90 min or with 5 µM Pac for 24 h (and mock controls).

Scale bar = 100 µm. **b**, Boxplot representation of GA signaling activity (Mock, n = 1738 nuclei examined over eight independent seedlings; 10 µM GA_3 for 90 min, n = 1326 nuclei examined over eight independent seedlings; 5 µM Pac for 24h, n = 1910 nuclei examined over eight independent seedlings) measured in *pRPS5a::qmRGA* seedling root tips grown in the same conditions as in (***a***)**,** indicated by different colors. **d**, Boxplot representation of GA signaling activity (Mock, n = 2044 nuclei examined over ten independent seedlings; 10 µM GA_3 for 90 min, n = 1602 nuclei examined over nine independent seedlings; 5 µM Pac for 24h, n = 1790 nuclei examined over nine independent seedlings) measured in *pRPS5a::qRGA*^{m3} seedling root tips grown in the same conditions as in (***c***)**,** indicated by different colors. **f**, Boxplot representation of GA signaling activity (Mock, n = 1874 nuclei examined over 9 independent seedlings; 10 µM GA_3 for 90 min, n=1782 nuclei examined over nine independent seedlings; 5 µM PAC for 24h, n=1750 nuclei examined over nine independent seedlings) measured in *pRPS5a::qRGA*^{m4} seedling root tips grown in the same conditions as in (***e***)**,** indicated by different colors. (**b,d,f**) Center lines show the medians and box limits indicate the 25th and 75th percentiles. Whiskers indicate minima and maxima as determined by R software. *P* values are from two-sided Kruskal-Wallis tests. The experiment was repeated twice with similar results. Scale bar = 100 µm.

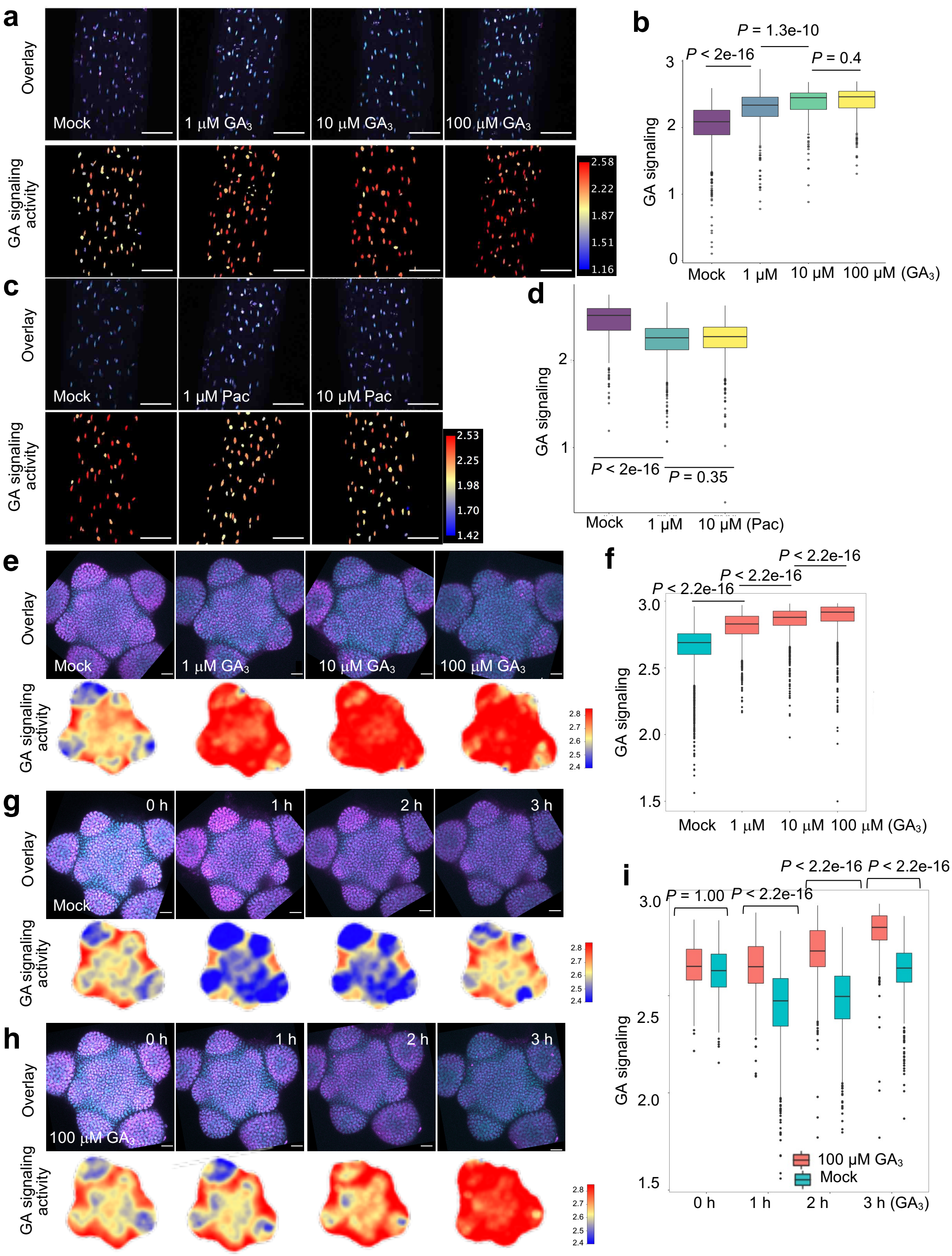

Supplementary Figure 4. Dose and temporal responses of *qmRGA* to exogenous GA or Pac treatment in seedling hypocotyls and the SAM.

a-d, Overlay of VENUS and TagBFP maximum intensity projection (upper row), and corresponding heatmap representation of GA signaling activity (lower row) in hypocotyls of 5-d-old *pUBQ10::qmRGA* seedlings treated with 1, 10 and 100 µM GA_3 for 90 min (**a**), or with 1 and 10 µM Pac for 24 h (**c**) and mock controls. **b**, Boxplot representation of GA signaling activity (Mock, n = 655 nuclei examined over eight

independent seedlings; 1 μM GA_3, n = 589 nuclei examined over nine independent seedlings; 10 μM GA_3, n = 489 nuclei examined over eight independent seedlings; 100 μM GA_3, n = 440 nuclei examined over eight independent seedlings) measured in *pUBQ10::qmRGA* seedling hypocotyls grown in the same conditions as in (**a**), indicated by different colors. *P* values are from two-sided Kruskal-Wallis tests. **d**, Boxplot representation of GA signaling activity (Mock, n = 664 nuclei examined over ten independent seedlings; 1 μM Pac, n = 621 nuclei examined over ten independent seedlings; 10 μM Pac, n=733 nuclei examined over ten independent seedlings) measured in *pUBQ10::qmRGA* seedling hypocotyls grown in the same conditions as in (**c**), indicated by different colors. *P* values are from two-sided Kruskal-Wallis tests. The experiment was repeated twice with similar results. **e-f**, Overlay of VENUS and TagBFP maximum intensity projection (upper row) and corresponding heatmap representation of GA signaling activity (lower row) of *pRPS5a::qmRGA* SAMs treated for 3 h with 1 μM, 10 μM and 100 μM GA_3, and mock controls (e). Boxplot representation of GA signaling activity is shown in (f). For Mock, n = 18816 nuclei examined over eight independent plants; for 1 μM GA_3, n = 6836 nuclei examined over three independent plants; for 10 μM GA_3, n = 7363 nuclei examined over three independent plants; for 100 μM GA_3, n = 6229 nuclei examined over three independent plants. *P* values are from two-sided Krustal-Wallis tests with Dunn's all pair rank comparison test. The experiment was repeated twice with similar results. **g-i**, Overlay of VENUS and TagBFP maximum intensity projection (g, h, upper rows) and corresponding heatmap representation of GA signaling activity (g, h, lower rows) of *pRPS5a::qmRGA* SAM treated with 100 μM GA_3 for 1 h, 2 h and 3 h (red boxes), and mock controls (blue boxes). Boxplot representation of GA signaling activity is shown in (i) For 0 h GA_3, n = 2450 nuclei examined over one independent plant; for 1 h GA_3, n = 2373 nuclei examined over one independent plant; for 2 h GA_3, n = 2056 nuclei examined over one independent plant; for 3 h GA_3, n = 2054 nuclei examined over one independent plant. The time course analysis upon GA treatment (h,i) was performed using 100 μM GA_3 in order to maximize the response of the sensor. *P* values are from two-sided Krustal-Wallis tests with Dunn's all pair rank comparison test. The

experiment was repeated twice with similar results. For all the boxplots, center lines show the medians and box limits indicate the 25th and 75th percentiles. Whiskers indicate minima and maxima as determined by R software. Scale bars = 100 μm (a,c), 20 μm (e, g, h).

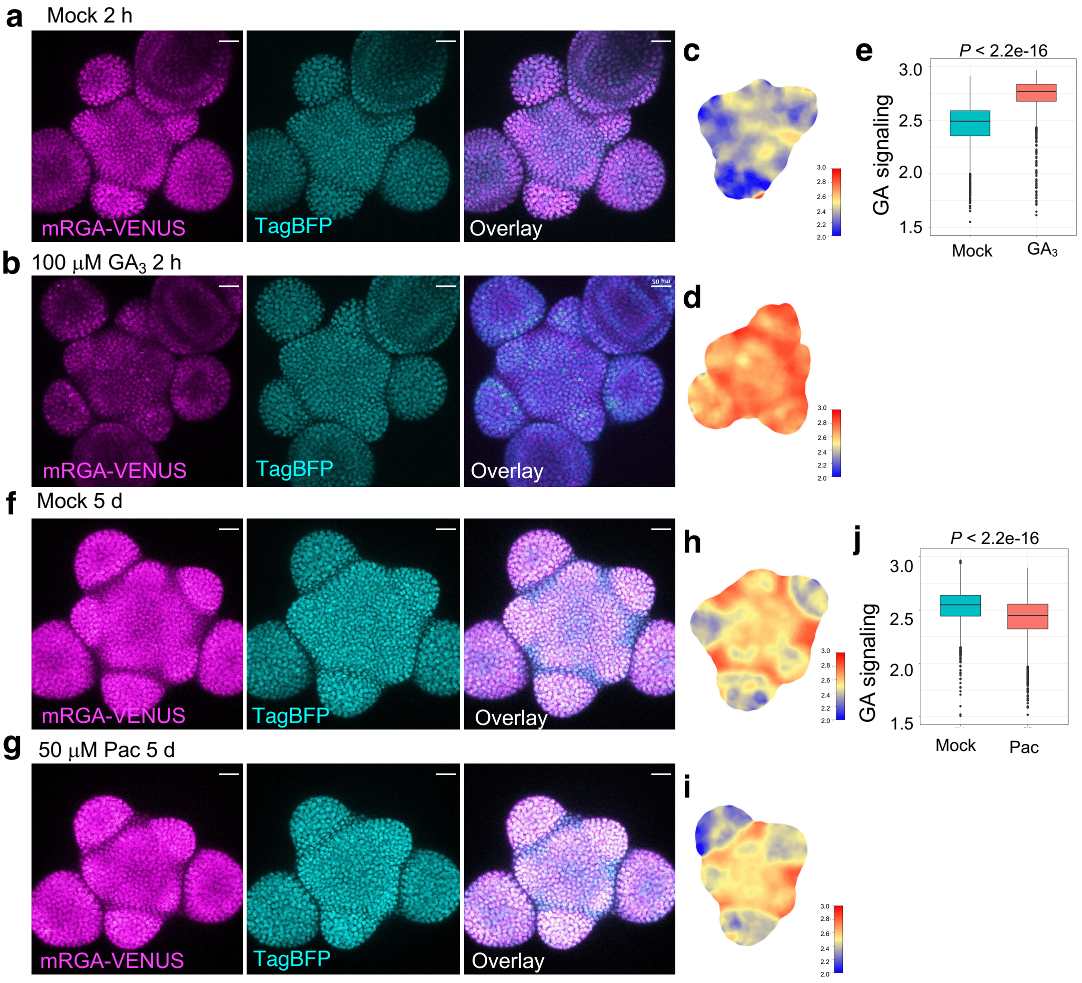

Supplementary Figure 5. Responses of *pRPS5a::qmRGA* to exogenous GA or Pac treatment in the SAM.

a-e, Fluorescence signals of mRGA-VENUS, TagBFP and overlay in *pRPS5a::qmRGA* SAM, 2 hours after a treatment with 100 µM GA_3 (b) and mock controls (a). (c, d) Heatmap representations of GA signaling activity. The older floral primordia were removed digitally. (e) Boxplot representations of GA signaling activity in SAM. For Mock, n = 6996 nuclei examined over five independent plants; for 2 h GA_3, n = 7011 nuclei examined over five independent plants. *P* value is from two-sided Wilcoxon rank-sum test. The experiment was repeated twice with similar results. **f-i**, Fluorescence signals of mRGA-VENUS, TagBFP and overlay in *pRPS5a::qmRGA* SAM, 5 days after a treatment with 50 µM Pac (g) and mock (f). (h, i) Heatmap representations of GA signaling activities from (f, g). Older floral primordia were removed digitally. (j) Boxplot representation of GA signaling activity in SAM (Mock,

n = 8150 nuclei examined over four independent plants; 5 d Pac, n = 11255 nuclei examined over seven independent plants). *P* value is from two-sided Wilcoxon rank-sum test. The experiment was repeated twice with similar results. For all the boxplots, center lines show the medians and box limits indicate the 25th and 75th percentiles. Whiskers indicate minima and maxima as determined by R software. Scale bars = 20 μm.

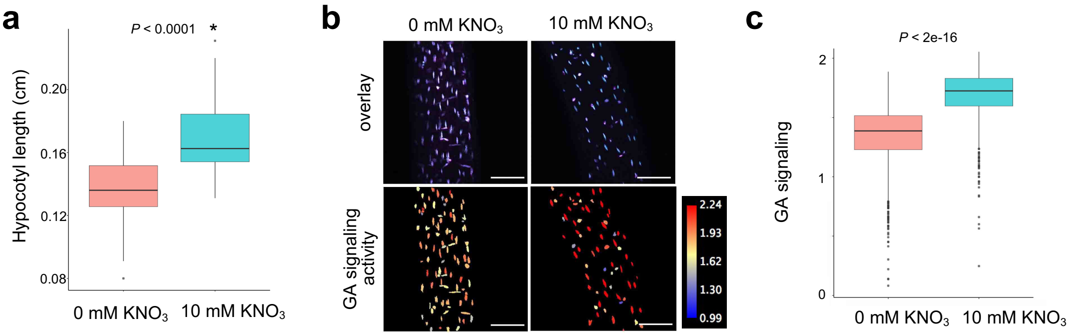

Supplementary Figure 6. Response of qmRGA to changes in endogenous GA levels in seedling hypocotyls.

a, Boxplot representations of the hypocotyl length of 6-d-old *pUBQ10::qmRGA* seedlings grown on nitrate-deficient conditions (0 mM KNO_3, n = 26 biological independent seedlings) and on 10 mM KNO_3 (n = 24 biological independent seedlings), indicated by different colors. Center lines show the medians and box limits indicate the 25th and 75th percentiles. Whiskers indicate minima and maxima as determined by R software. *P* value is from two-tailed Welch t-test. **b**, **c**, Overlay of mRGA-VENUS and TagBFP maximum intensity projection, and corresponding heatmap representation of GA signaling activity in hypocotyls of 5-d-old *pUBQ10::qmRGA* seedlings grown on nitrate-deficient conditions and on 10 mM KNO_3. **c**, Boxplot representation of GA signaling activity (0 mM KNO_3, n = 1037 nuclei examined over eleven independent seedlings; 1 µM GA_3, n = 589 nuclei examined over nine independent seedlings; 10 mM KNO_3, n = 410 nuclei examined over eight independent seedlings) measured in *pUBQ10::qmRGA* seedling hypocotyls grown in the same conditions as in (**b**), indicated by different colors. Center lines show the medians and box limits indicate the 25th and 75th percentiles. Whiskers indicate minima and maxima as determined by R software. *P* value is from two-sided Wilcoxon rank-sum test. The experiment was repeated twice with similar results. Scale bar = 100 µm.

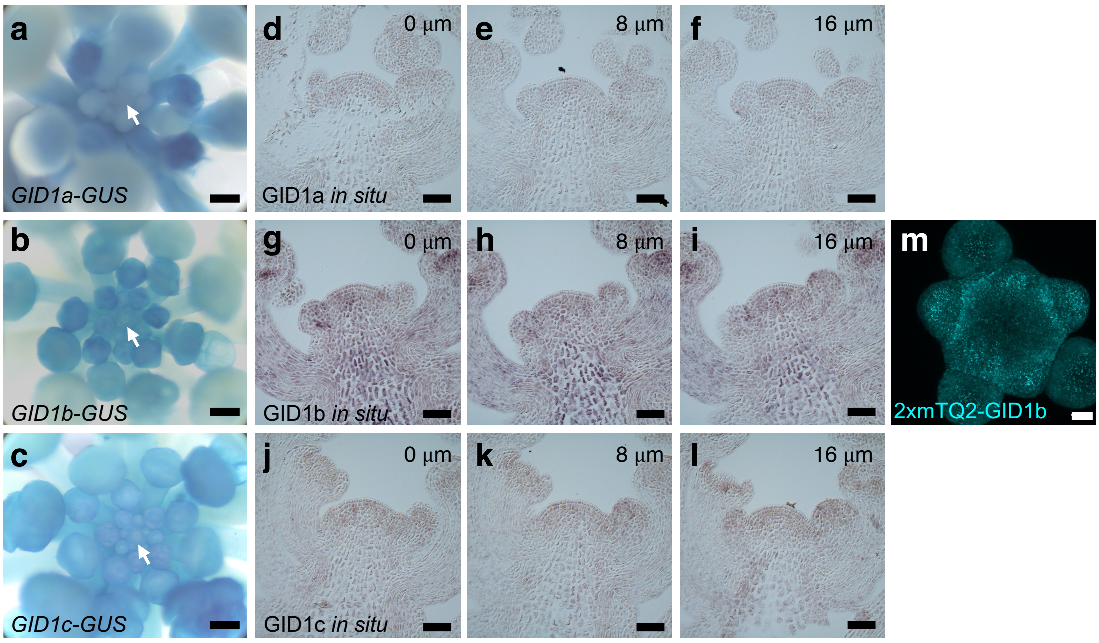

Supplementary Figure 7. Expression patterns of GA receptors in the SAM.

a-c, Expression patterns of *GID1a*, *GID1b* and *GID1c* in the SAM of *pGID1a::GID1a-GUS* (**a**), *pGID1b::GID1b-GUS* (**b**) and *pGID1c::GID1c-GUS* (**c**) adult plants. The white arrows highlight the different extends of blue staining in the SAM regions of different marker lines. The experiment was repeated twice with similar results. **d-l**, *In situ* localization of *GID1a* (**d-f**), *GID1b* (**g-i**) and *GID1c* (**j-l**) mRNA in inflorescence apex. Three consecutive sections are shown for each. The experiment was repeated twice with similar results. **m**, Maximum intensity projection showing the expression of *pGID1b::2xmTQ2-GID1b*. The experiment was repeated twice with similar results. Scale bars = 200 µm (a-c) and 20 µm (d-m).

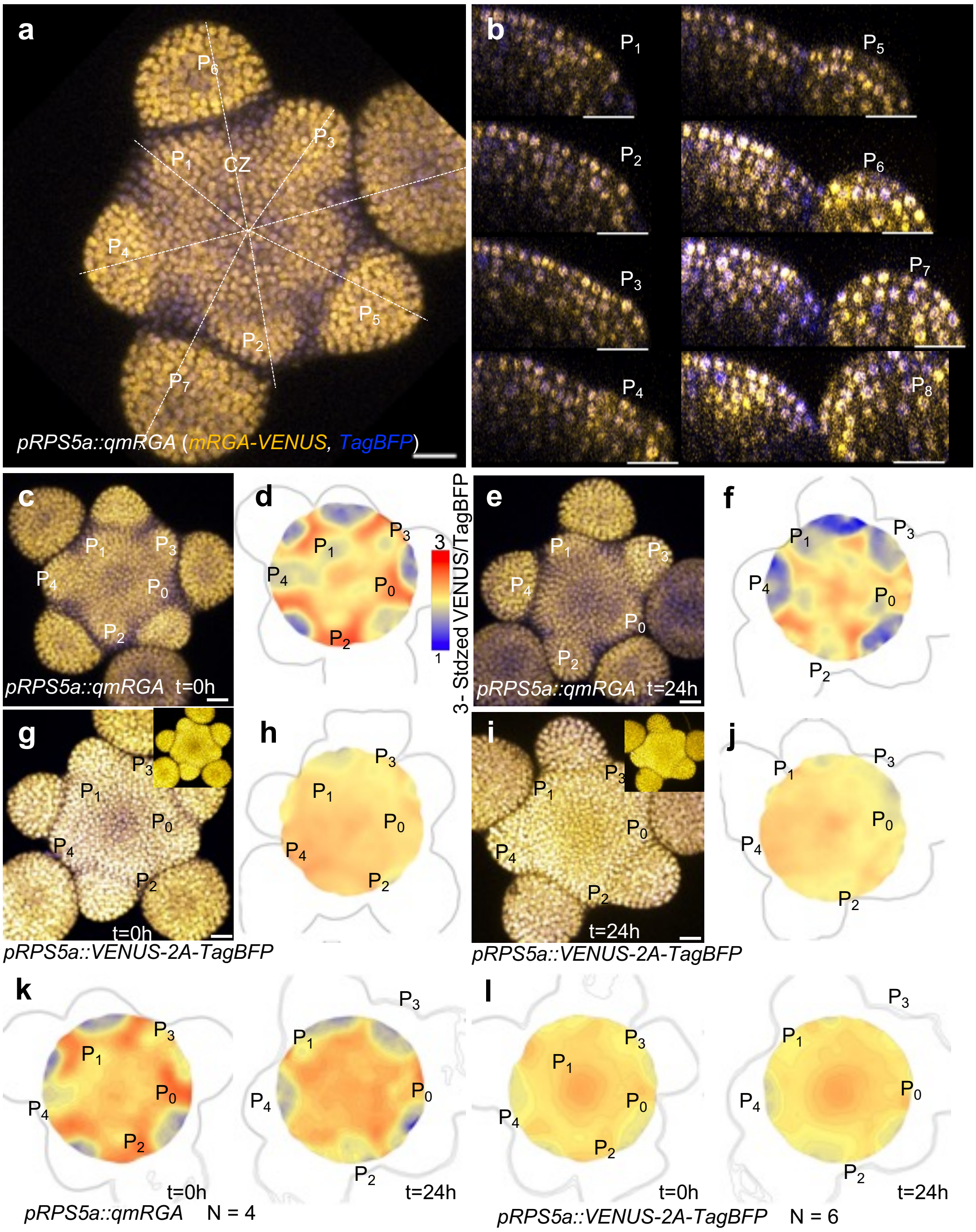

Supplementary Figure 8. Analysis of the qmRGA pattern in the SAM over time and comparison to the pattern of the pRPS5a::VENUS-2A-TagBFP control line. **a**, Maximum intensity projection showing the expression of *pRPS5a::qmRGA* in the SAM as in Fig 4a. CZ, central zone. **b**, Signals for mRGA-VENUS (yellow) and TagBFP-NLS (blue) in digital longitudinal sections through the center of CZ and primordia P_1 to P_8 along the white dotted lines indicated in (**a**). The experiment was repeated twice with similar results. **c-l**, Comparison of the expression pattern of *pRSP5a::qmRGA* and *pRPS5a::VENUS-2A-TagBFP* over time in the SAM. One

representative *pRSP5a::qmRGA* SAM and one representative *pRPS5a::VENUS-2A-TagBFP* SAM are shown at 0 h and 24 h in (**c-f**) and (**g-j**) respectively: maximum projections of the overlaid yellow (VENUS) and blue (TagBFP) are shown in (**c,e,g,i**) and the corresponding quantification are shown as heatmaps in (**d,f,h,j**). A heatmap representation of L1 GA signaling activity averaged from four aligned SAMs is shown in (**k**) at 0 h and 24 h. A heatmap representation of L1 *pRPS5a::VENUS-2A-TagBFP* fluorescence ratio distribution averaged from six aligned SAM is shown in (**l**) at 0 h and 24 h. Note that to allow for the comparison of the two lines, the values of ratios obtained for each image were standardized by dividing them by their mean and the heatmaps represent the distribution of 3 - standardized (stdzed) VENUS / TagBFP as indicated in the color scale in (**d**). The color scale is the same for all heatmaps. The experiment was repeated twice with similar results. Scale bars = 20 μm.

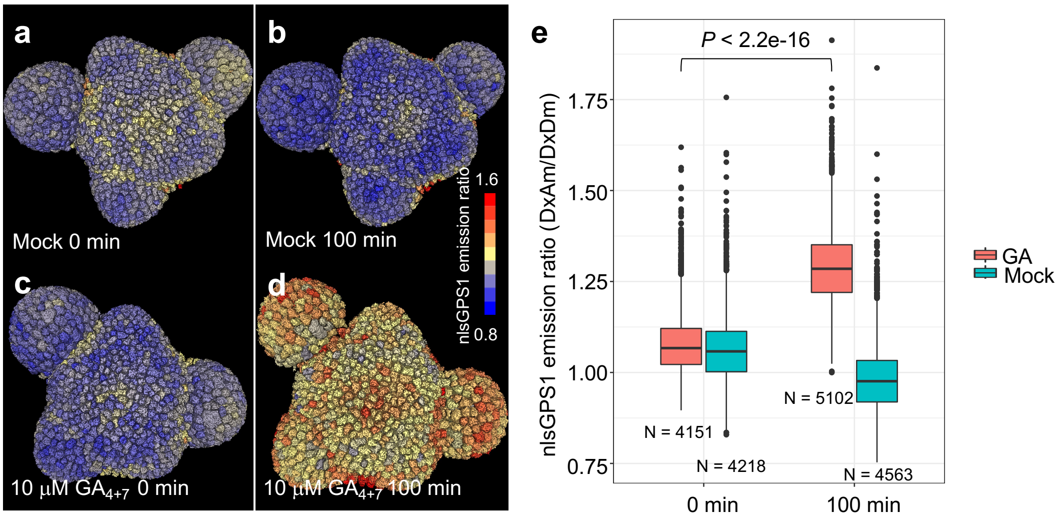

Supplementary Figure 9. Response of nlsGPS1 to GA treatment in the SAM.

a-d, 3D visualization of nlsGPS1 expression in the SAM before (**a**, **c**) and after (**b**, **d**) mock (**a**, **b**) and 10 μM GA_{4+7} treatment (**c**, **d**). The experiment was repeated twice with similar results. **e**, Boxplot representation of nlsGPS1 emission ratios from nuclei analyzed in three SAMs. The number of nuclei (N) from three independent plants is indicated. *P* value is from two-sided Wilcoxon rank-sum test.

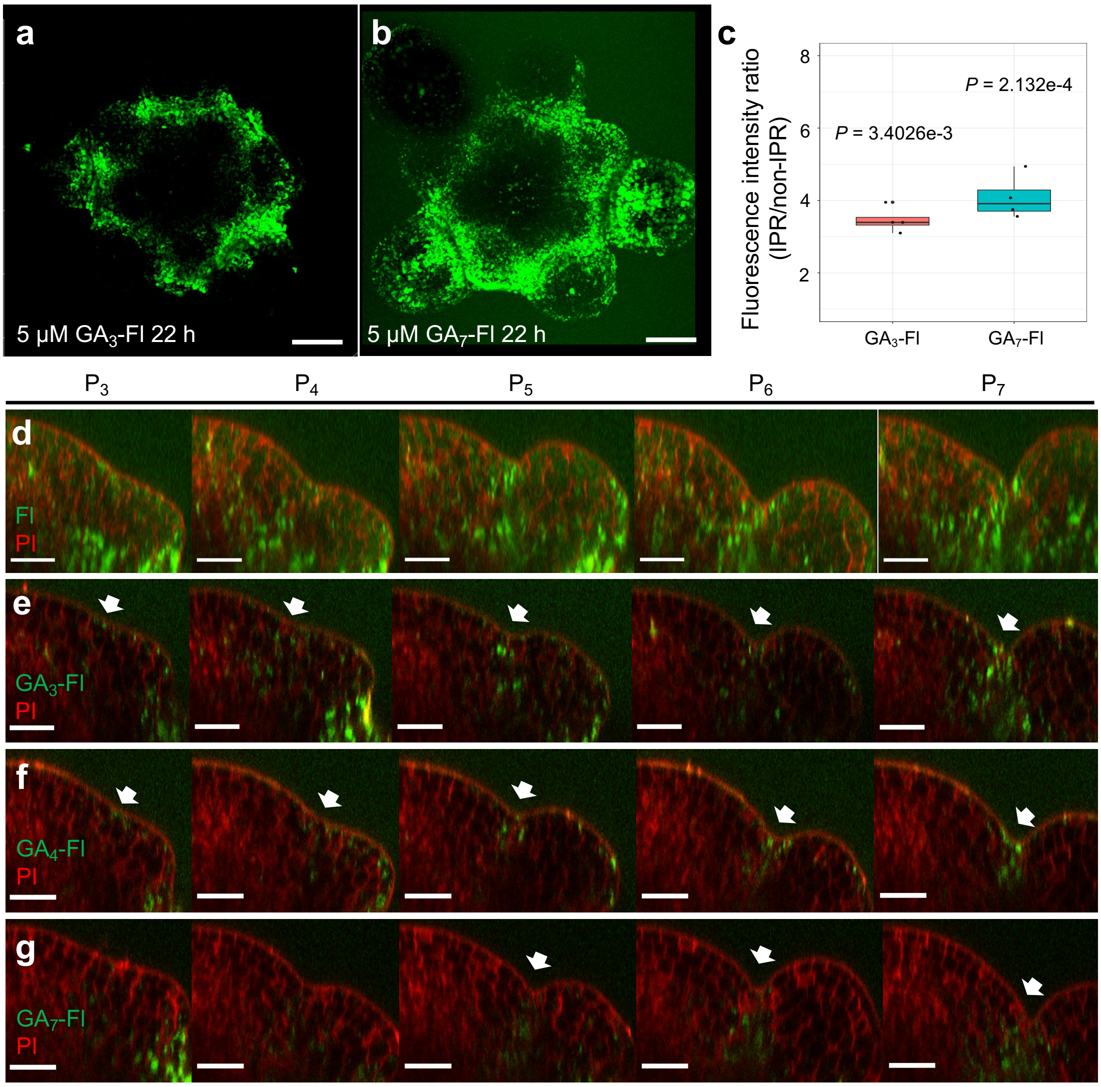

Supplementary Figure 10. Fluorescence distribution in SAMs treated with Fl and GA_3-, GA_4- and GA_7-Fl.

a-b, Fluorescence distribution in wild-type (L*er*) SAMs treated with GA_3-Fl (**a**) and GA_7-Fl (**b**). **c**, Ratio of average fluorescence intensity in the IPR to that in the non-IPR (excluding primordia) after GA_3-Fl (n = 4 independent plants) and GA_7-Fl (n = 4 independent plants) treatment in the SAM, compared to Fl treatment (n = 4 independent plants; Fig 4f, h). Center lines show the medians and box limits indicate the 25th and 75th percentiles. Whiskers indicate minima and maxima as determined by R software. *P* values are from one-way ANOVA with Turkey's test for multiple comparisons of means. The experiment was repeated twice with similar results. **d-g,** Digital longitudinal sections through the center of CZ and primordia P_3 to P_7 of L*er* SAMs treated with 5 μM fluorescein (**d**), GA_3-Fl (**e**), GA_4-Fl (**f**), or GA_7-Fl (**g**) (green) and

stained with propidium iodide (PI, red). The experiment was repeated twice with similar results. Scale bars = 20 μm.

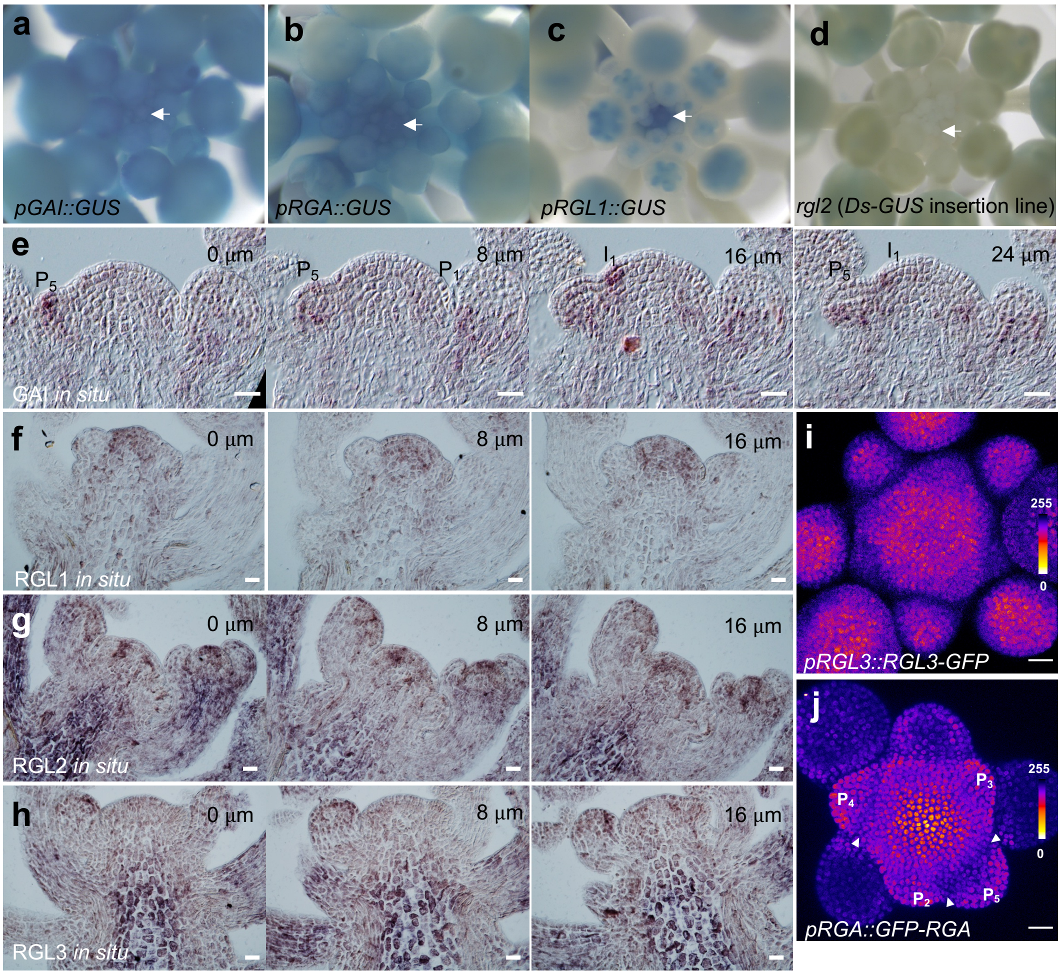

Supplementary Figure 11. Expression patterns of DELLA-encoding genes and accumulation DELLA proteins in the SAM.

a-d, GUS signal in the shoot apex of *pGAI::GUS* (**a**), *pRGA::GUS* (**b**), *pRGL1::GUS* (**c**) and *rgl2-5* (a promoter trap GUS line) mutant (**d**). The experiment was repeated twice with similar results. **e-h**, *In situ* hybridization serial sections through a shoot apex using probes of GAI (**e**), RGL1 (**f**), RGL2 (**g**) and RGL3 (**h**). The experiment was repeated twice with similar results. **i-j**, Intensity heatmaps of maximum intensity-projection of *pRGL3::RGL3-GFP* (**i**) and *pRGA::GFP-RGA* (**j**) in the SAM. Arrowheads mark the regions with lower GFP-RGA expression. The experiment was repeated twice with similar results. Scale bars = 20 µm.

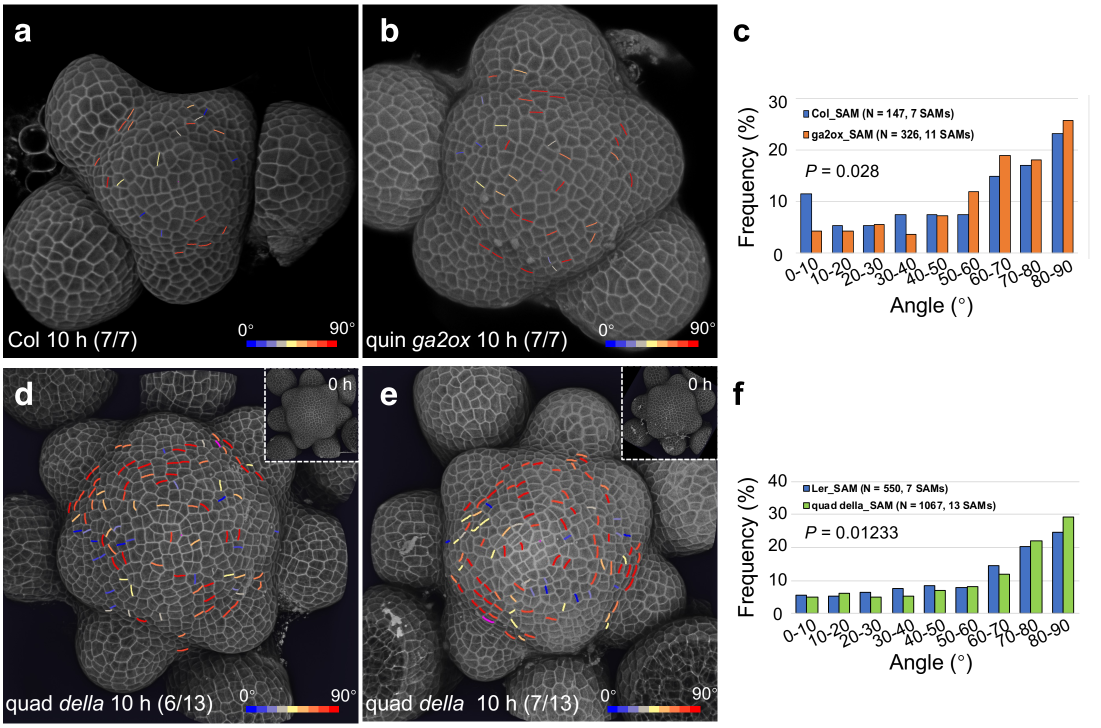

Supplementary Figure 12. Cell division plane orientation distribution in the SAM of quintuple *ga2ox* and quadruple *della* mutants.

a-c, 3D reconstruction of confocal stacks showing the epidermis of PI-stained SAMs from wild-type plants (Col-0, **a**) and from the quintuple *ga2ox* mutant (**b**). New cell walls formed in the SAM (but not in primordia) over a 10 h time course are shown and colored according to their angle values. (**c**) Comparison of frequency distributions of division plane orientation between cells located in the entire SAM of Col (n = 147 division planes over 7 independent plants) and quintuple *ga2ox* mutants (n = 326 division planes over 11 independent plants). *P*-value is from two-sided Kolmogorov-Smirnov test. The experiment was repeated three times with similar results. **d-f**, 3D reconstruction of confocal stacks showing the epidermis of the PI-stained SAMs of quadruple *della* mutant. New cell walls formed in the SAM (but not in primordia) in 10 h are shown and colored according to their angle values. (**d**) Representative results of 6 out of 13 SAMs, and (**e**) of the remaining 7 SAMs where a higher frequency of transverse cell divisions could be detected. Inserts are images at 0 h. (**f**) Comparison of frequency distributions of division plane orientation between cells located in the entire SAM of L*er* (n = 550 division planes over 7 independent plants) and quadruple *della* mutant (n = 1067 division planes over 13 independent plants). *P*

value is from two-sided Kolmogorov-Smirnov test. The experiment was repeated twice with similar results.

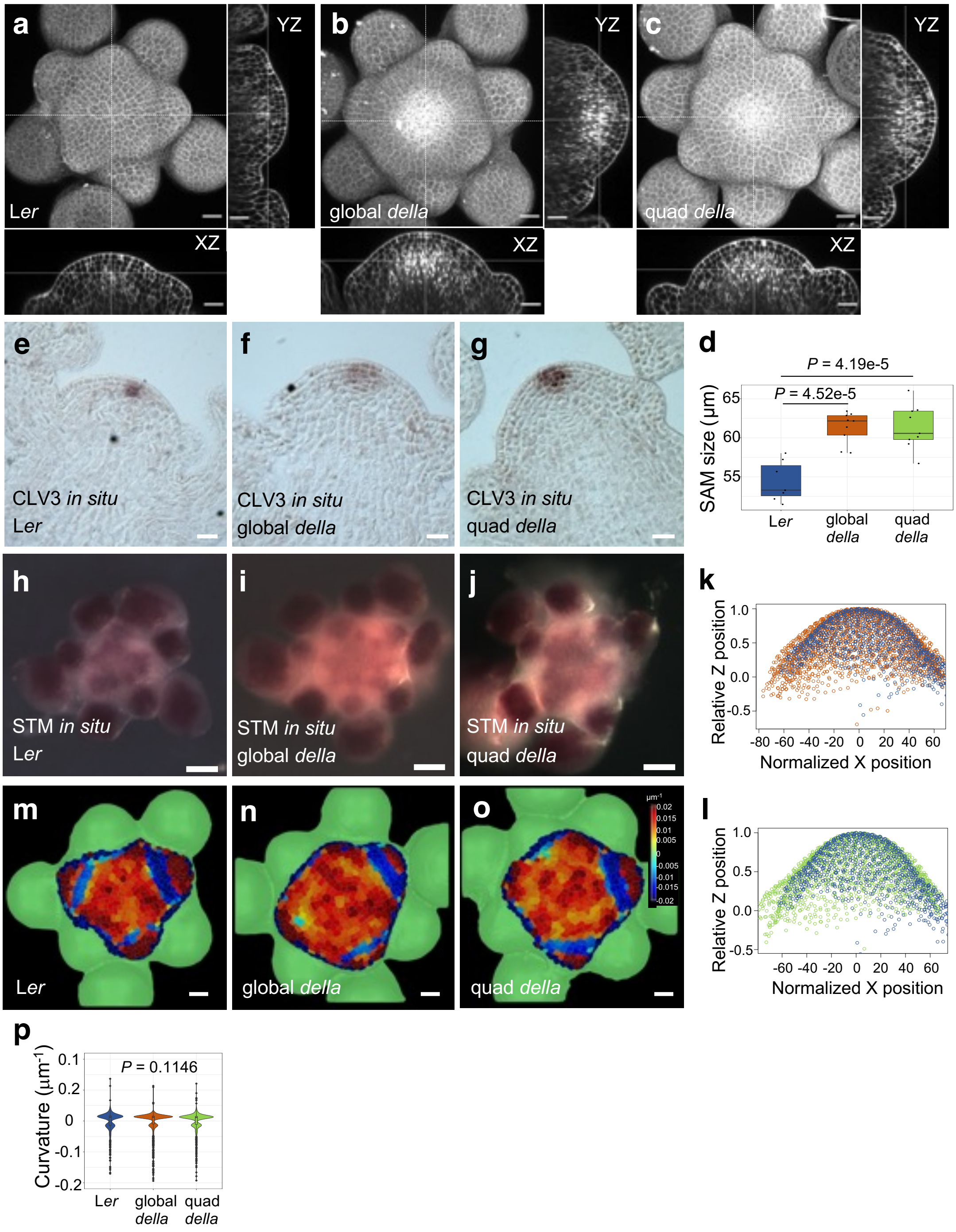

Supplementary Figure 13. Analysis of SAM shape and gene expression in global and quadruple *della* mutants.

a-c, Maximum projection (**a-c**) and orthogonal views (XZ dimension, lower panels**;** YZ dimension, left panels) of confocal stacks of PI-stained SAMs of L*er* (**a**), global *della* (**b**) and quadruple *della* mutant (**c**). **d**, Comparison of SAM size between L*er* (n = 7 independent plants), global (n = 9 independent plants) and quadruple (n = 9 independent plants) *della* mutants. Center lines show the medians and box limits

indicate the 25th and 75th percentiles. Whiskers indicate minima and maxima as determined by R software. *P* values are from one-way ANOVA with Tukey's multiple comparisons of means. The experiment was repeated twice with similar results. **e-g**, RNA *in situ* hybridization of *CLV3* using sections of L*er* (**e**), global *della* (**f**) and quadruple *della* mutant (**g**) SAMs. The experiment was repeated twice with similar results. **h-j**, Whole-mount RNA *in situ* hybridization of *STM* of L*er* (**h**), global *della* (**i**) and quadruple *della* mutant (**j**) SAMs. The experiment was repeated twice with similar results. **k-l**, XZ point plots of L1 nuclei of L*er* (blue dots in **k** and **l**), global *della* (orange in **k**) and quadruple *della* (green in **l**) showing the tissue-level curvature of the SAM. **m-p**, Heatmaps of cellular curvature of L1 cells in the SAM (including P_4) of L*er* (**m**), global *della* (**n**) and quadruple *della* (**o**). Statistics are shown in (**p**). For L*er*, n = 873 cells over two independent plants; for global *della*, n = 1888 cells over four independent plants; for quadruple *della*, n = 1437 cells over three independent plants. Center lines of the embedded boxplots show the medians and box limits indicate the 25th and 75th percentiles. Whiskers indicate minima and maxima as determined by R software. *P* values are from two-sided Kruskal-Wallis tests. Scale bars = 20 μm (a-c, e-g, m-o) and 50 μm (h-j).

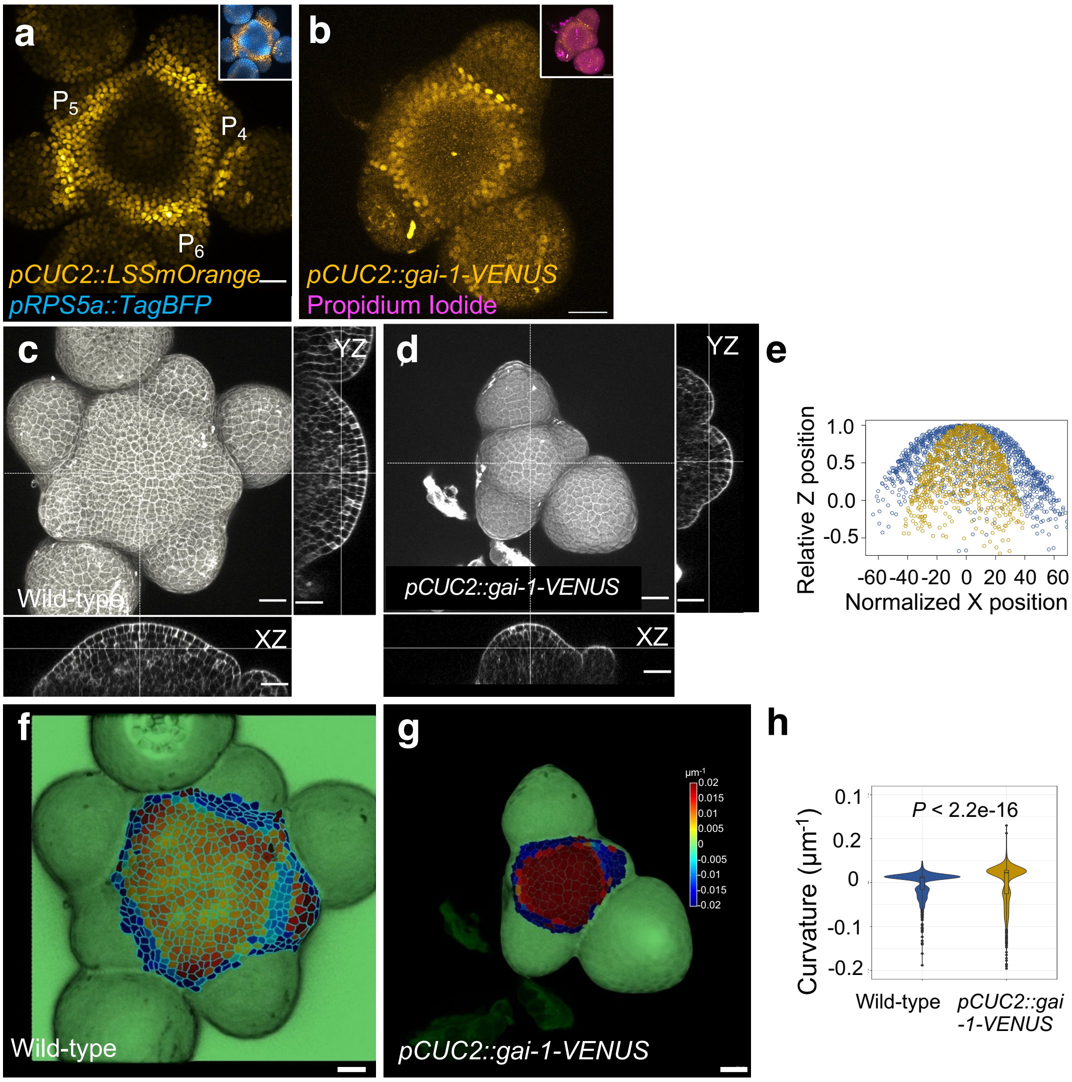

Supplementary Figure 14. Analysis of transgene expression and SAM shape in *pCUC2::gai-1-VENUS* transgenic plants.

a, Maximum projection view of the SAM of *pCUC2::LSSmOrange-NLS* reporter line. The insert shows the same image superimposed with the expression of *pRPS5a::TagBFP-NLS*. **b**, Maximum projection view of the SAM of *pCUC2::gai-1-VENUS* transgenic plants. The same image overlaid with propidium iodide (PI) staining was shown in the upper-right corner. The experiment was repeated twice with similar results. **c-d**, Maximum projection and orthogonal views (XZ dimension, lower panels**;** YZ dimension, left panels) of PI-stained SAM of *pCUC2::gai-1-VENUS* (**c**) transgenic plant and its wild-type control (**d**). The experiment was repeated twice with similar results. **e**, XZ point plots of L1 nuclei of wild-type (blue) and *pCUC2::gai-1-VENUS* transgenic plants (yellow) showing the

tissue-level curvature of the SAM. **f-h**, Heatmaps of cellular curvature of L1 cells in the SAM of wild-type (**f**) and *pCUC2::gai-1-VENUS* (**g**) transgenic plants. Statistics are shown in (**h**). For wild-type, n = 1206 cells over three independent plants; for *pCUC2::gai-1-VENUS*, n = 875 cells over four independent plants. *P* value is from two-sided Wilcoxon rank-sum test. The experiment was repeated twice with similar results. Center lines of the embedded boxplots show the medians and box limits indicate the 25th and 75th percentiles. Whiskers indicate minima and maxima as determined by R software. Scale bars = 20 μm.

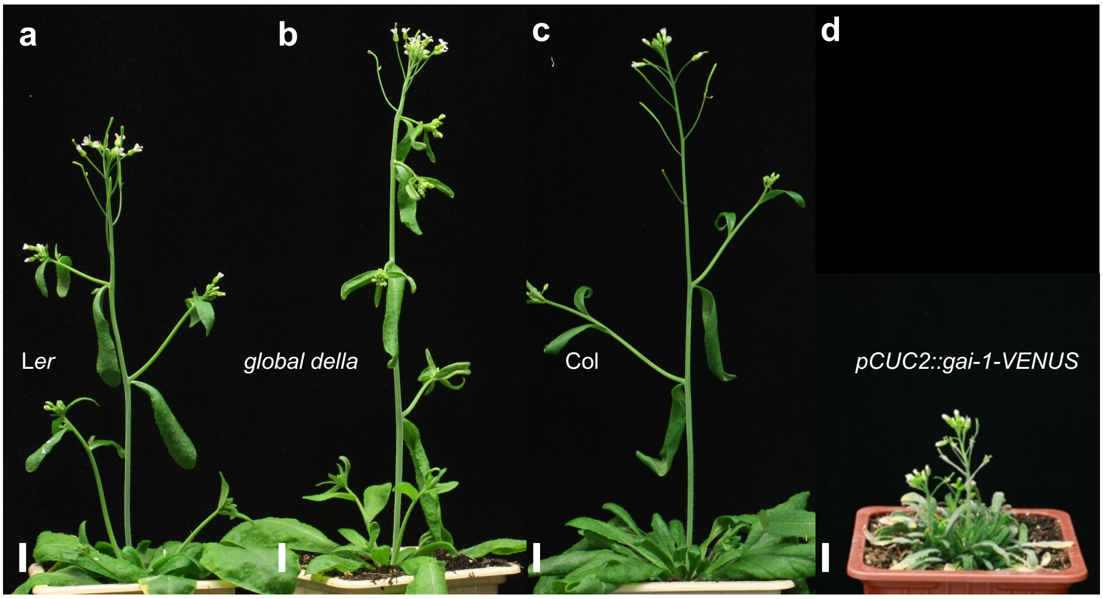

Supplementary Figure 15. Altered plant height in global *della* mutant and the *pCUC2::gai-1-VENUS* transgenic plants.

a-b, Representative images of a 30-d-old wild-type (L*er*, **a**) and a global *della* mutant plant (**b**) grown under long-day conditions. **c-d,** Representative images of a 37-d-old wild-type (Col, **c**) and a 56-d-old *pCUC2::gai-1-VENUS* transgenic plants (**d**) grown under long-day conditions. Scale bar = 1 cm.

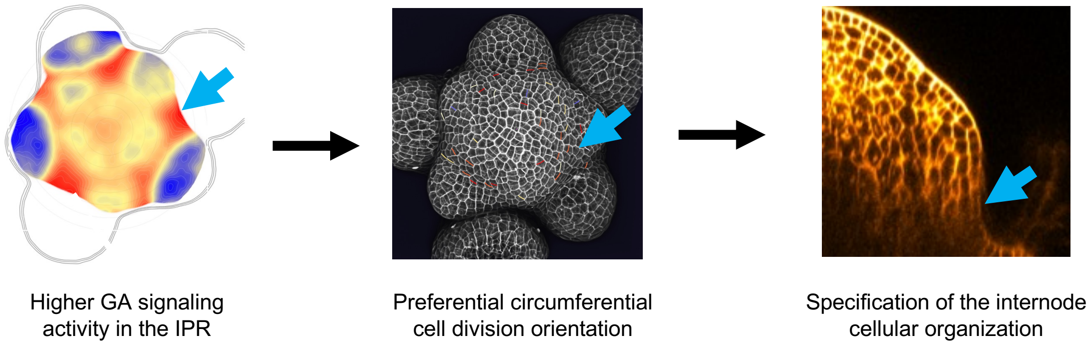

Supplementary Figure 16. Model of the role of GA signaling in internode specification.

Higher levels of GA signaling activity in the IPR specify the cellular organization of internodes in the SAM via the regulation of the orientation of cell division.

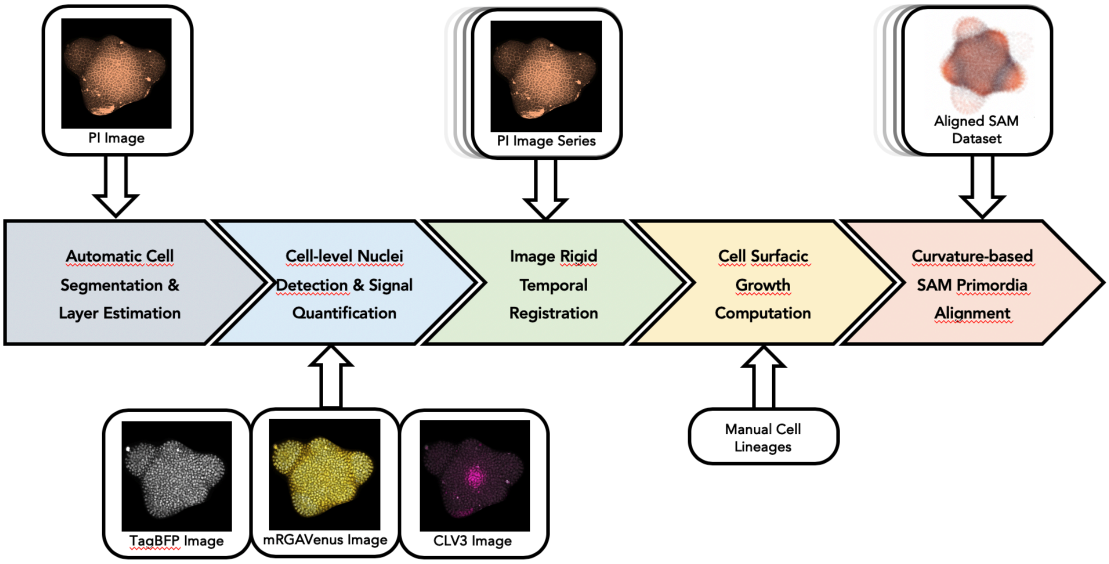

Supplementary Figure 17. Automatic quantification pipeline for the time-lapse microscopy images.

To obtain quantitative data from the images produced under the microscope, various sequential processing steps need to be performed, from the extraction of the relevant objects (cells regions based on a cell wall marker channel, nuclei positions with their different channel intensity values) to the geometrical characterization and the spatio-temporal registration of the tissues, to finally get a complete, aligned and consistent dataset gathering all the imaged meristems.

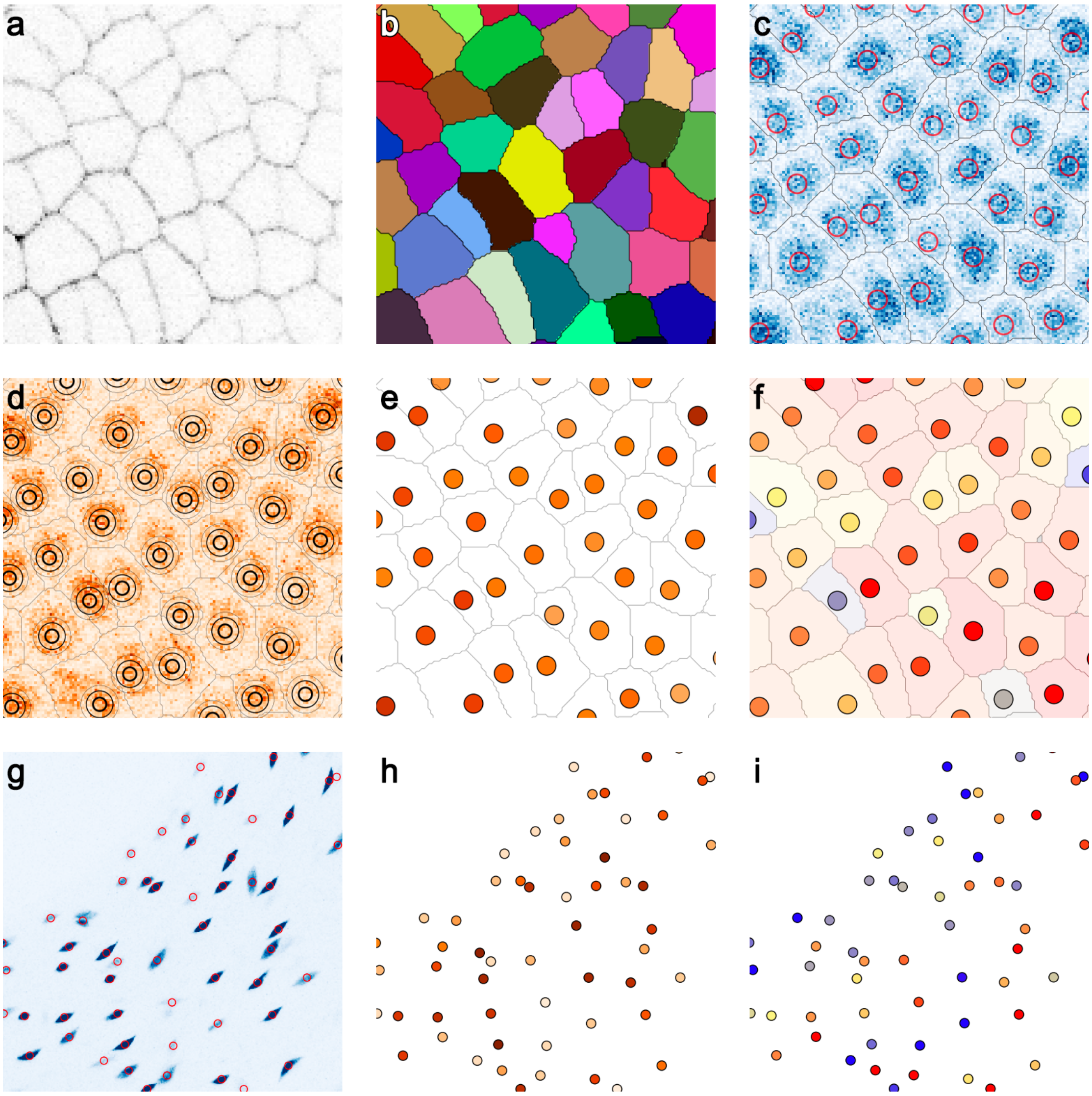

Supplementary Figure 18. Automatic cell nuclei detection and ratiometric signal quantification.

a-b, The PI channel (**a**, slice view) provides a reference for the position of the cell walls in the 3D confocal stack, which is used to perform a watershed segmentation (**b**, surface view) that assigns each voxel of the image to a cell region, identified by a unique label. **c**, The TagBFP (BFP) channel (**c**, slice view) is used to detect precisely the position of cell nuclei through a Gaussian scale-space transform, using the cell regions from the segmented image to limit the detection to one nucleus per cell. **d-f**, Signals such as the one provided by the mRGA-VENUS (mRGAV) channel (**d**, slice view) are quantified as a Gaussian-weighted average of the intensity values around each detected nucleus, associating each cell with a signal value (**e**). To obtain the level of GA signaling, the BFP signal is quantified in the same way to be able to compute a

ratio of the two signals. The quantified value for the GA signaling (**f**) is obtained through the formula GA = 3 − mRGAV/BFP. **g-i**, In the case of hypocotyl images, the nuclei detection is performed on the BFP channel (**g**, max-intensity projection) with specific parameter values and without the cell region constraint, signals are then quantified similarly from their respective channel (**h**) and the GA ratiometric value computed using the same formula (**i**).

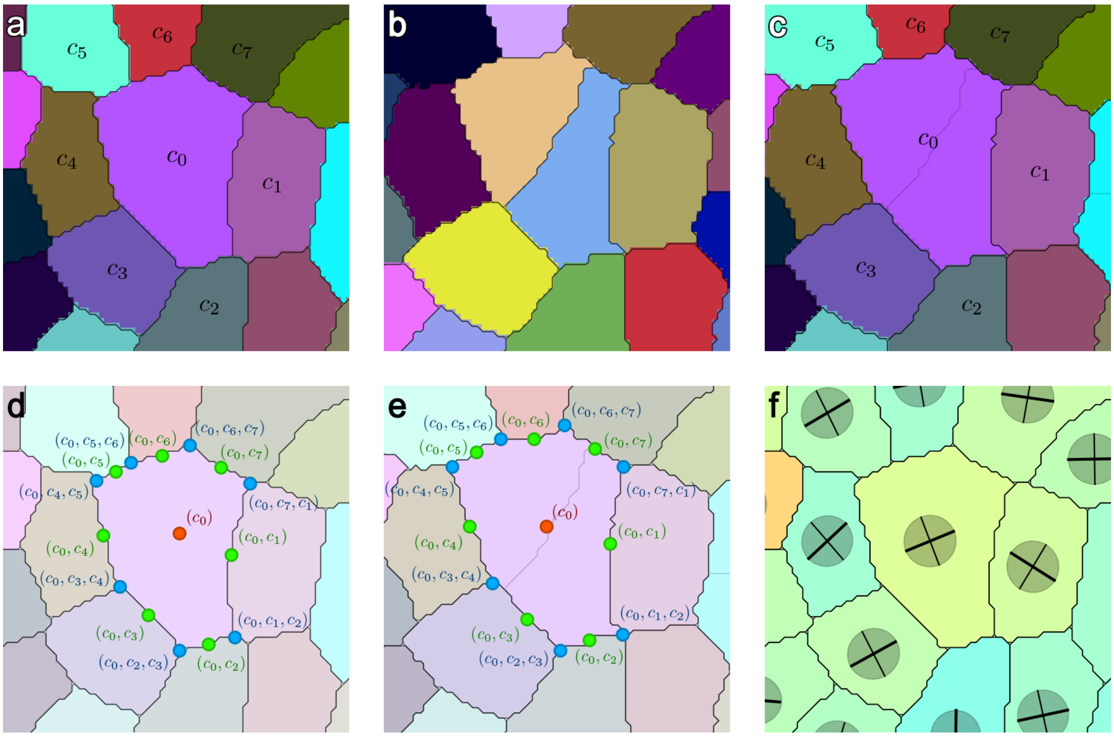

Supplementary Figure 19. Surfacic strain estimation using cell landmarks.

a-b, View of the surface of segmented images of the same tissue at two consecutive time points. Each cell carries a label identifier that is used to manually define lineages of L1 cells. **c**, The lineage information allows to relabel the image of the second time point using the cell labels of the first one. **d-e**, Landmarks representing the junctions between two or more labels in the image are extracted on both the first image (**d**) and the relabelled second image (**e**). The tuples of cell labels associated with each landmark point allow to pair them between the two consecutive time points. **f**, In each cell, the linear least-squares estimation of the deformation results in the determination of a surfacic strain tensor, defining two strain values and two orthogonal growth directions.

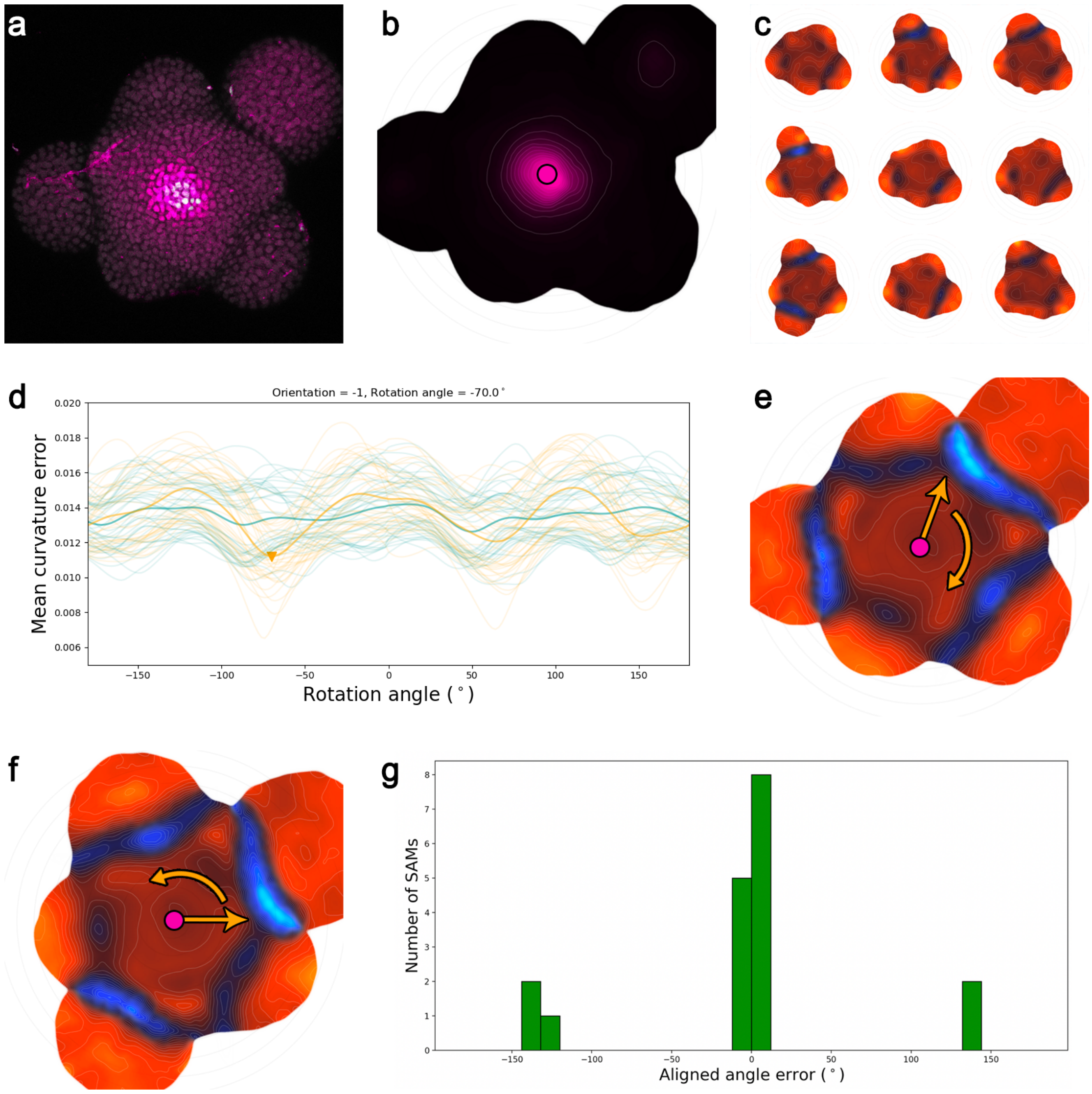

Supplementary Figure 20. SAM alignment onto a reference set of curvature maps. **a-b**, The CLV3 image channel (**a**, max-intensity projection), quantified at the level of each cell nucleus, is used to locate precisely the center of the SAM. It is done using a continuous 2D map (**b**), which is computed using the registered positions of all L1 nuclei of the sequence. This allows to center the nuclei point cloud, and subsequently to correct its tilting by estimating the main vertical axis of the SAM. **c-e**, Next, to identify the direction of the lastly initiated primordium, we rely on a database of aligned SAM point clouds from [1] on which curvature has been computed. This provides a set of reference 2D curvature maps (**c**, subset of the database) that are used to determine an optimal angle and orientation (**d**, error curves obtained for each reference map, $o = 1$ in green and $o = -1$ in orange) by minimizing the error with the 2D curvature map of the considered SAM (**e**). **f**, This process allows to align the

SAM in a common reference frame where inter-individual comparison is possible. **g**, The evaluation of this alignment procedure on the reference database shows that orientation is correctly predicted and that the correct alignment angle is retrieved in 72% of the cases. All the remaining cases show an error corresponding to a shift of ±1 divergence angle between two consecutive organs.

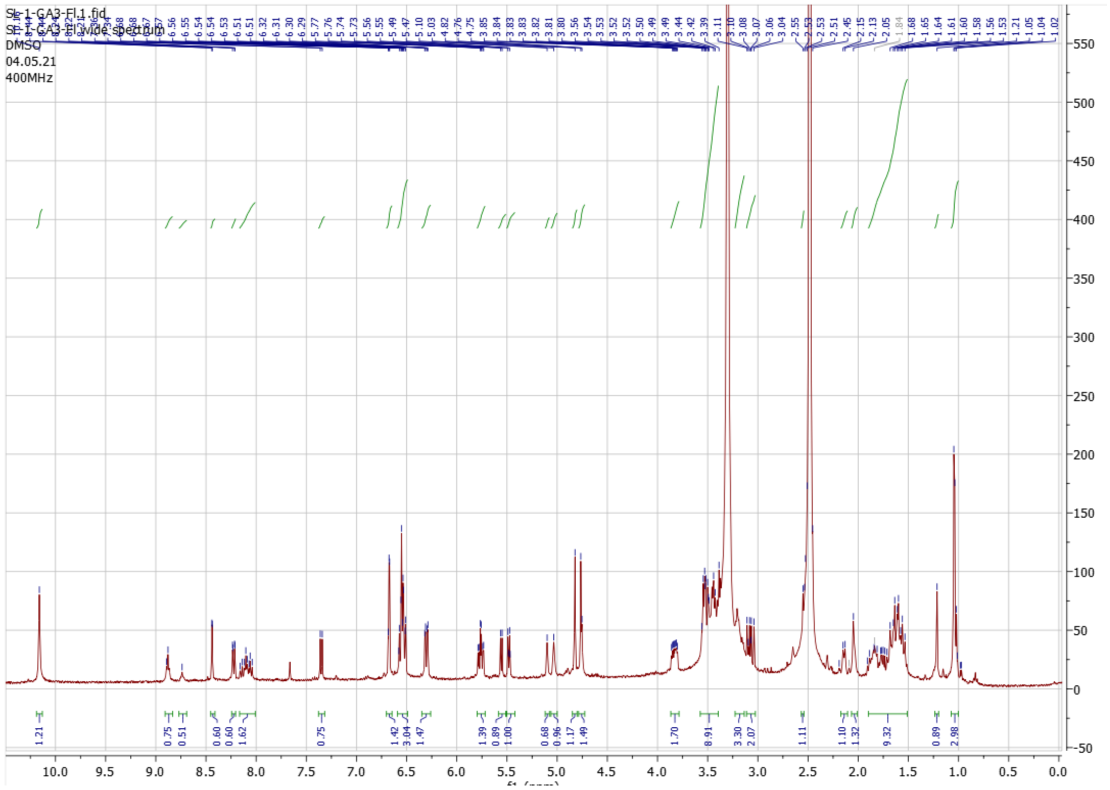

Supplementary Figure 21. ^{1}H-NMR spectrum of GA$_3$-Fl.

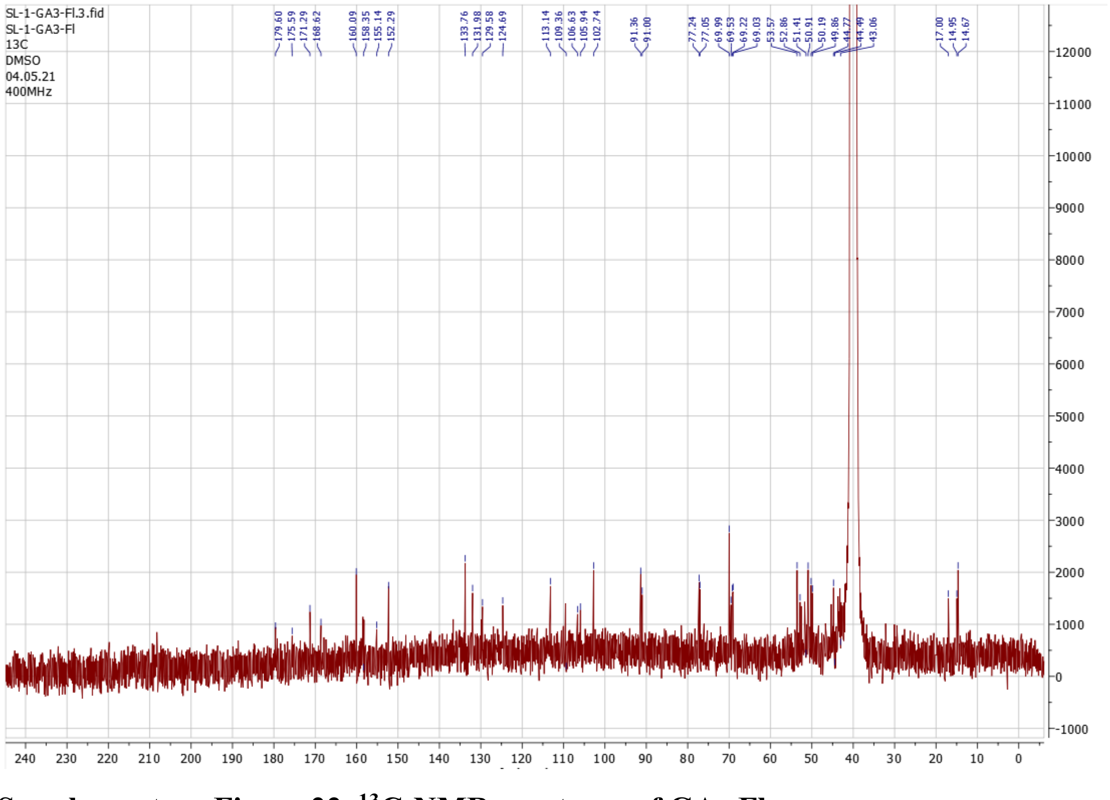

Supplementary Figure 22. ^{13}C-NMR spectrum of GA$_3$-Fl.

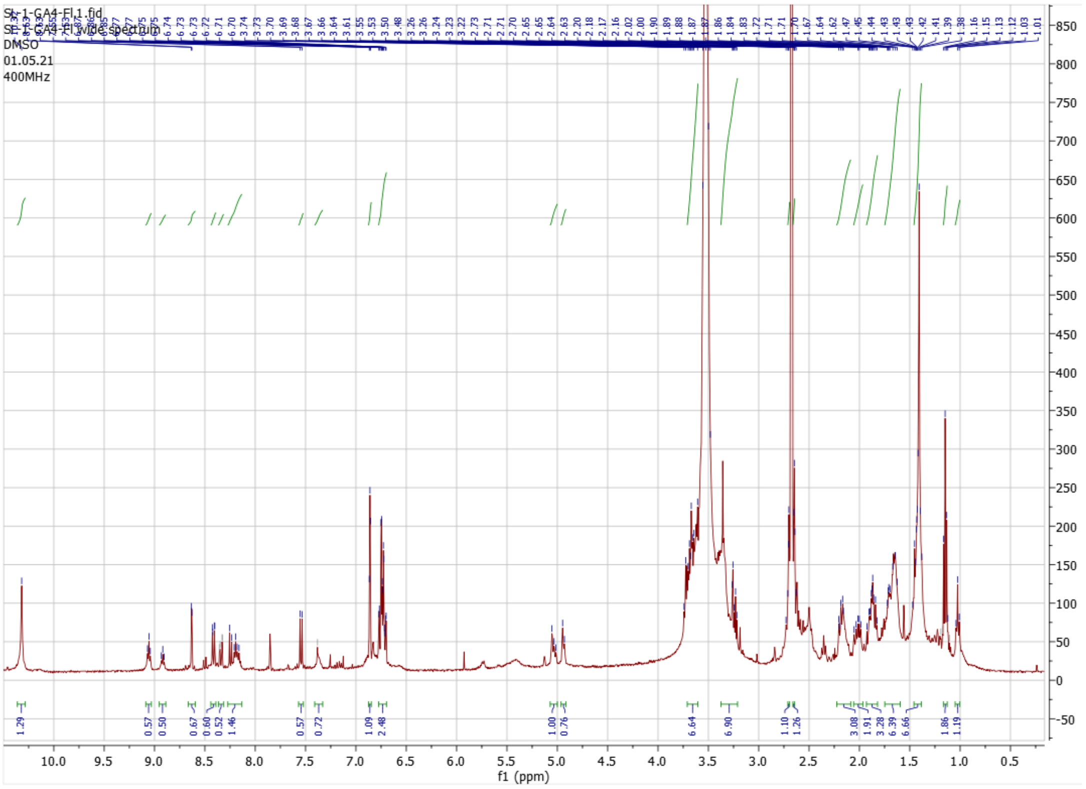

Supplementary Figure 23. ^{1}H-NMR spectrum of GA_4-Fl.

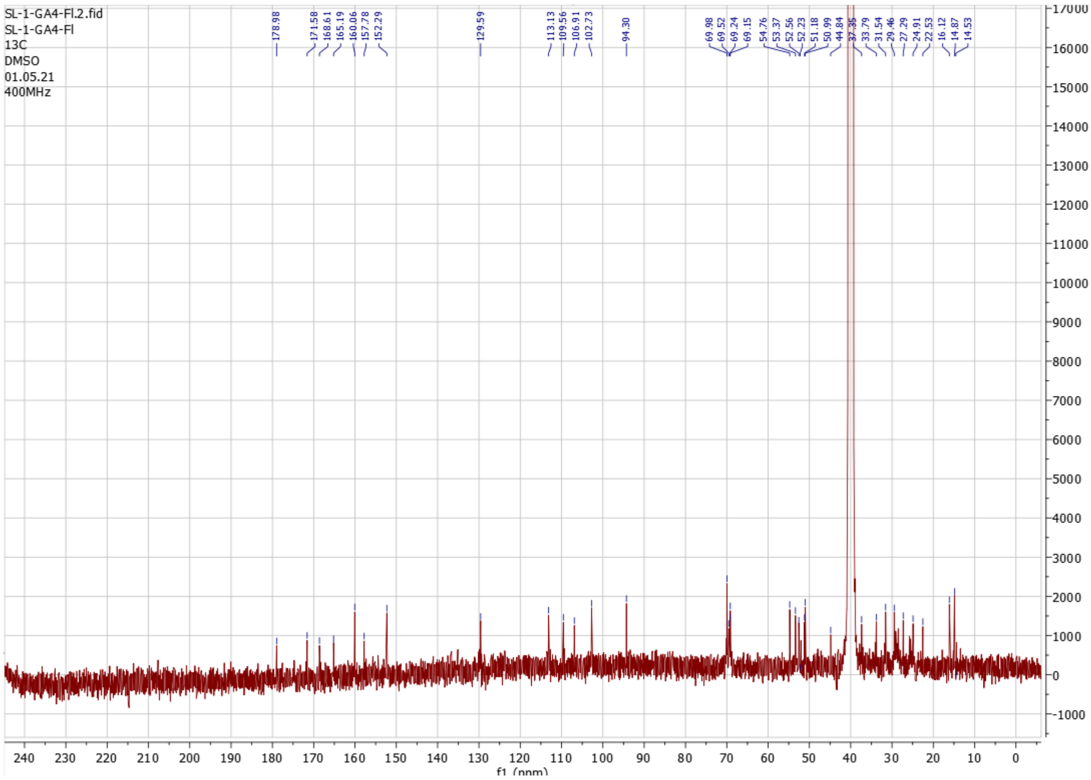

Supplementary Figure 24. ^{13}C-NMR spectrum of GA_4-Fl.

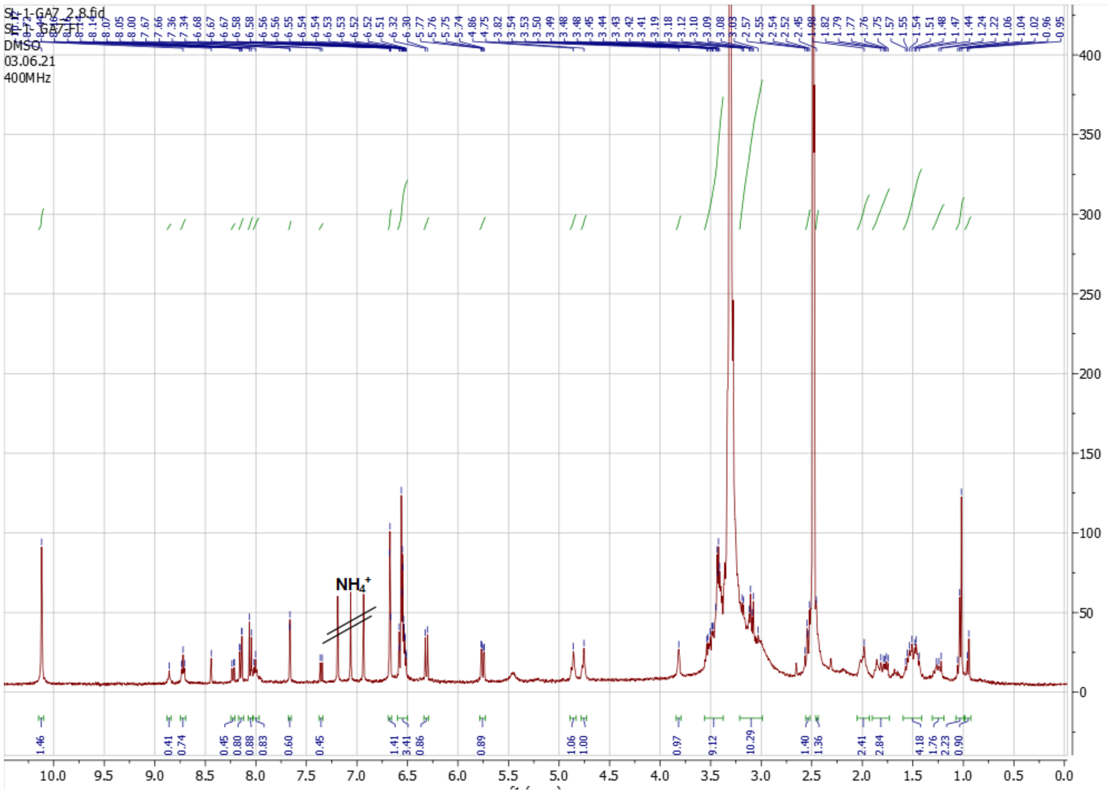

Supplementary Figure 25. ^{1}H-NMR spectrum of GA$_7$-Fl.

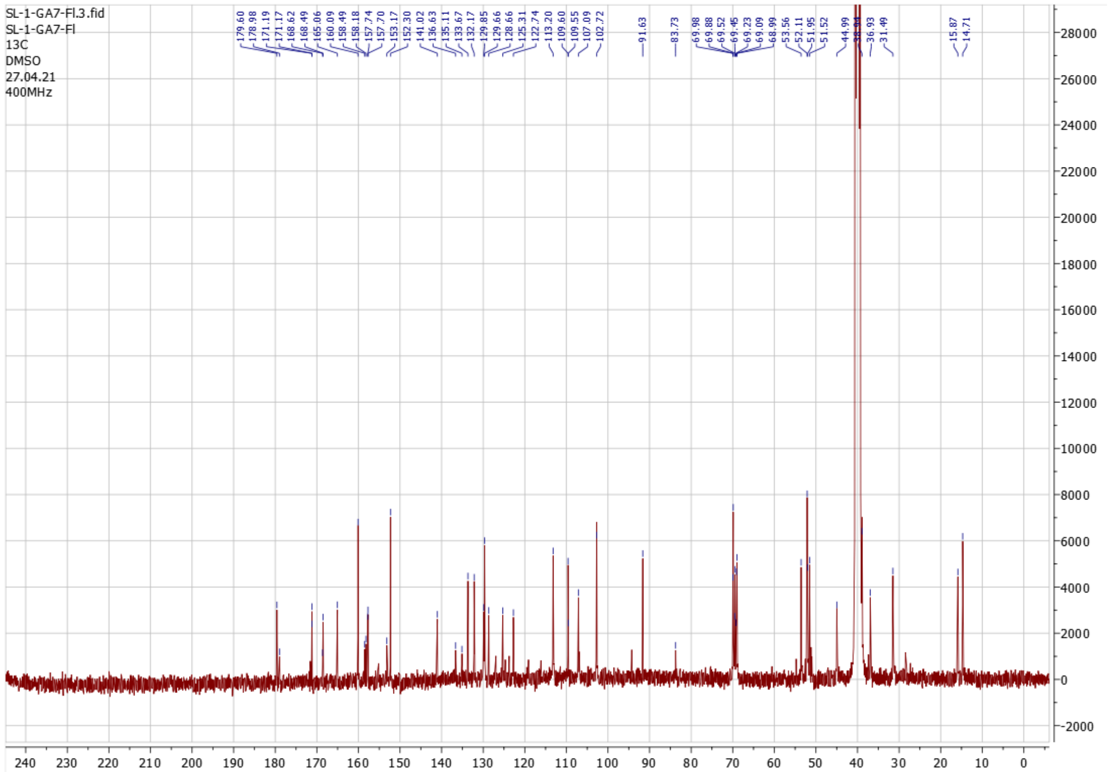

Supplementary Figure 26. ^{13}C-NMR spectrum of GA$_7$-Fl.

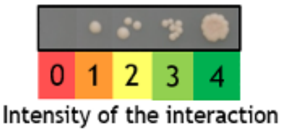

AGI	TF Name	Interaction in -LWH			Interaction in -LWH + 1mM 3AT		
		Ø	M5RGA	M5RGA^{m2}	Ø	M5RGA	M5RGA^{m2}
No gene	Control	0	0	0	0	0	0
At4g11880	AGL14	0	3	0	0	0	0
At5g13790	AGL15	0	3	0	0	0	0
At5g65050	AGL31	0	3	0	0	0	0
At5g23260	AGL32/ABS/TT16	0	4	0	0	1	0
At2g28550	TOE1/RAP2.7	0	1	0	0	0	0
At5g60100	APRR3	0	1	0	0	0	0
At1g09530	bHLH8/EN100/PIF3	0	2	0	0	0	0
At5g53420	CO-like/CCT?	0	3	0	0	0	0
At2g22750	bHLH18	0	1	0	0	0	0
At3g43430	C3HC4	0	1	0	0	0	0
At5g10030	bZIP57/OBF4/TGA4	0	1	0	0	0	0
At1g12980	DRN/ESR1/ERF089	0	3	0	0	2	0
At2g22770	bHLH20/EN27/NAI1	0	3	0	0	1	0
At3g61630	CRF6/ERF067	0	2	0	0	0	0
At5g53950	CUC2/ANAC098	0	2	0	0	0	0
At3g15510	NAC2/ANAC056	0	3	0	0	1	0
At3g29035	NAC3/ANAC059/ORS1	0	3	0	0	0	0
At2g24430	ANAC038	0	1	0	0	0	0
At3g18400	ANAC058	0	1	0	0	0	0
At1g32870	ANAC13	0	3	0	0	0	0
At5g18270	ANAC087	0	2	0	0	1	0
At3g21330	bHLH87/EN121	0	2	0	0	0	0
At5g67060	bHLH88/EN118/HEC1	0	3	0	0	2	0
At1g06170	bHLH89/EN24	0	3	1	0	0	0
At2g31210	bHLH91/EN25	0	3	0	0	0	0
At1g69170	SPL6	0	1	0	0	0	0
At3g15270	SPL5	0	1	0	0	0	0
At5g52510	SCL8	0	1	0	0	0	0
At1g67260	TCP1	0	2	1	0	0	0
At4g18390	TCP2	0	3	0	0	3	0
At1g53230	TCP3	0	4	2	0	4	0
At5g60970	TCP5	0	2	0	0	0	0
At1g58100	TCP8	0	2	0	0	1	0
At2g31070	TCP10	0	4	1	0	4	0
At3g45150	TCP16	0	3	3	0	1	0
At1g35560	TCP23	0	3	3	0	1	0
At5g08330	TCP21	0	1	0	0	0	0
At5g51910	TCP19	0	2	2	0	2	0
At1g69180	CRC/CRU1/CRU3	0	1	0	0	0	0
At2g43000	JUB1/ANAC042	0	4	1	0	1	0
At3g12910	NAC/NAM	0	2	0	0	1	0
At5g11060	KNAT4	0	1	0	0	0	0
At3g61150	HDG1	0	1	0	0	0	0
At5g64810	WRKY51	0	2	0	0	0	0
At2g44410	Zn finger	0	1	0	0	0	0
At4g17490	ERF6	0	2	0	0	0	0
At2g40340	ERF48/DREB2C	0	1	0	0	0	0
At4g32980	ATH1	0	1	0	0	0	0
At2g23760	SAW2/BLH4	0	4	0	0	3	0
At5g65790	MYB68	0	4	0	0	3	0
At2g01060	PHL7	0	1	0	0	0	0
At3g23240	ERF092/ERF1/ERF1B	0	1	0	0	0	0
At4g26930	MYB97	0	3	0	0	0	0
At5g21120	EIL2	0	3	0	0	2	0
At2g31380	STH	0	1	0	0	0	0
At4g16750	ERF039	0	1	0	0	0	0
At5g25810	ERF040/TINY	0	1	0	0	0	0
At1g27050	HB54	0	2	0	0	0	0
At5g66700	HB-8/HB-53	0	1	1	0	1	0
At2g28910	CXIP4	0	1	0	0	0	0
At4g21040	DOF4.3	0	1	0	0	0	0

At4g01350	DC1	0	1	0	0	0	0
At2g02080	ATIDD4	0	3	0	0	4	0
At4g03250	HDZIP	0	1	0	0	0	0
At4g17460	HAT1	0	4	0	0	1	0
At3g04420	ANAC048	0	1	0	0	1	0
At3g02310	AGL4/SEP2	0	1	0	0	0	0
At1g17590	NFYA8	0	2	0	0	0	0
At5g06839	bZIP65/TGA10	0	3	0	0	0	0
At1g25340	MYB116	0	2	0	0	0	0
At5g62320	MYBCU15/MYB99	0	1	0	0	0	0
At1g17770	SUVH7/SDG17/SET17	0	1	0	0	0	0
At1g09540	MYB61	0	4	0	0	3	0
At4g24240	WRKY7	0	3	0	0	2	0
At1g69810	WRKY36	0	1	0	0	1	0
At5g24520	TTG1	0	1	0	0	0	0
At3g47620	TCP14	0	1	0	0	0	0
At1g69690	TCP15	0	1	0	0	0	0
At1g31630	AGL86	0	1	0	0	0	0
At5g26650	AGL36	0	2	0	0	0	0
At2g24430	ANAC038	0	1	0	0	0	0
At3g21175	GATA24/ZML1	0	1	0	0	0	0
At1g24625	ZFP7	0	1	0	0	1	0
At3g02150	TCP13	0	3	0	0	1	0
At2g45050	GATA2	0	1	0	0	0	0
At3g20310	ERF083/ERF7	0	2	1	0	1	0
At5g18560	PUCHI/ERF086	0	2	0	0	0	0
At1g49480	REM19/RTV1	0	1	1	0	0	0
At5g16560	KAN1	0	1	0	0	0	0
At5g05090	Homeodomain/G2-like?	0	3	0	0	2	0
At1g51140	bHLH122/EN70/ Flowering bHLH3/FBH3	0	4	0	0	3	0
At2g42280	Flowering bHLH4/ FBH4	0	2	0	0	0	0
At1g61660	bHLH112/EN64	0	4	0	0	4	0
At5g64750	ABR1/ERF111	0	3	0	0	2	0
At5g54070	HSFA9	0	1	0	0	0	0
At5g09460	bHLH143/EN129/AJAX3	0	1	0	0	0	0
At1g19210	ERF017	0	2	0	0	0	0
At5g17690	LHP1/TFL2	0	2	0	0	0	0
At4g13980	HSF12/HSFA5	0	1	0	0	1	0
At4g30935	WRKY32	0	3	1	0	2	0
At1g68920	bHLH49/EN82	0	1	0	0	0	0
At3g09600	RVE8/LCL5	0	2	0	0	0	0
At1g51600	GATA28/ZML2	0	2	0	0	0	0
At3g29035	NAC3/ANAC059/ORS1	0	3	0	0	1	0
At4g37750	ANT	0	3	0	0	1	0
At1g77200	ERF037	0	1	0	0	0	0
At3g57600	DREB2F	0	1	0	0	0	0
At2g28910	CXIP4	0	1	0	0	0	0
At1g31630	AGL86	0	2	0	0	0	0
At5g26650	AGL36	0	3	0	0	0	0
At5g29000	PHL1	0	3	0	0	0	0
At2g33880	HB-3/STIP/WOX9A	0	3	0	0	2	0

Supplementary Table 1. RGA^{m2} loses its ability to bind with RGA-interacting partners.

Extended pairwise Y2H interaction assays between RGA, mRGA and RGA-interacting transcription factors. The color code and numbers (0 to 5) indicate the strength of the interactions based on the size of the yeast colonies on selective media without leucine, tryptophan and adenine (-LWH) without or with 1 mM 3-aminotriazole (3AT). Empty pGBKT7 and pGADT7 vectors were included as negative controls.

	A. List of constructions used in this paper				
	**Plasmid name**	**Backbone**	**DONR vectors**		**Source**
**Sensor screening and development**	p35S::RGA-GFP	pB7WGF2	pDONR221 RGA		this study
	p35S::RGA^{m1}-GFP	pB7WGF2	pDONR221 RGA^{m1}		this study
	p35S::RGA^{m2}-GFP	pB7WGF2	pDONR221 RGA^{m2}		this study
	p35S::RGA^{m3}-GFP	pB7WGF2	pDONR221 RGA^{m3}		this study
	p35S::RGA^{m4}-GFP	pB7WGF2	pDONR221 RGA^{m4}		this study
	pRPS5a::qRGA	pB7m34GW	pDONR P4-P1R pRPS5a , pDONR221 RGA-VENUS , pDONR P2R-P3 2A-TagBFP-NLS		this study
	pUBQ10::qRGA	pB7m34GW	pDONR P4-P1R pUBQ10 , pDONR221 RGA-VENUS , pDONR P2R-P3 2A-TagBFP-NLS		this study
	pRPS5a::qmRGA	pB7m34GW	pDONR P4-P1R pRPS5a , pDONR221 mRGA-VENUS , pDONR P2R-P3 2A-TagBFP-NLS		this study
	pUBQ10:qmRGA	pB7m34GW	pDONR P4-P1R pUBQ10 , pDONR221 mRGA-VENUS , pDONR P2R-P3 2A-TagBFP-NLS		this study
	pRPS5a::RGA^{m1}-VENUS-2A-TagBFP-NLS	pB7m34GW	pDONR P4-P1R pRPS5a , pDONR221 RGA^{m1}-VENUS , pDONR P2R-P3 2A-TagBFP-NLS		this study
	pRPS5a::RGA^{m3}-VENUS-2A-TagBFP-NLS	pB7m34GW	pDONR P4-P1R pRPS5a , pDONR221 RGA^{m3}-VENUS , pDONR P2R-P3 2A-TagBFP-NLS		this study
	pRPS5a::RGA^{m4}-VENUS-2A-TagBFP-NLS	pB7m34GW	pDONR P4-P1R pRPS5a , pDONR221 RGA^{m4}-VENUS , pDONR P2R-P3 2A-TagBFP-NLS		this study
	pUBQ10::RGA^{m1}-VENUS-2A-TagBFP-NLS	pB7m34GW	pDONR P4-P1R pUBQ10 , pDONR221 RGA^{m1}-VENUS , pDONR P2R-P3 2A-TagBFP-NLS		this study
	pUBQ10::RGA^{m3}-VENUS-2A-TagBFP-NLS	pB7m34GW	pDONR P4-P1R pUBQ10 , pDONR221 RGA^{m3}-VENUS , pDONR P2R-P3 2A-TagBFP-NLS		this study
	pUBQ10::RGA^{m4}-VENUS-2A-TagBFP-NLS	pB7m34GW	pDONR P4-P1R pUBQ10 , pDONR221 RGA^{m4}-VENUS , pDONR P2R-P3 2A-TagBFP-NLS		this study
	p35S::d17RGA-VENUS	pB7m34GW	pDONR P4-P1R p35S , pDONR221 d17RGA , pDONR P2R-P3 VENUS		this study
	p35S::d17mRGA-VENUS	pB7m34GW	pDONR P4-P1R p35S , pDONR221 d17mRGA , pDONR P2R-P3 VENUS		this study
	pRPS5a::qd17RGA	pB7m34GW	pDONR P4-P1R pRPS5a , pDONR221 d17RGA , pDONR P2R-P3 2A-TagBFP-NLS		this study
	pUBQ10::qd17RGA	pB7m34GW	pDONR P4-P1R pUBQ10 , pDONR221 d17RGA , pDONR P2R-P3 2A-TagBFP-NLS		this study
	pRPS5a::qd17mRGA	pB7m34GW	pDONR P4-P1R pRPS5a , pDONR221 d17mRGA , pDONR P2R-P3 2A-TagBFP-NLS		this study
	pUBQ10::qd17mRGA	pB7m34GW	pDONR P4-P1R pUBQ10 , pDONR221 d17mRGA , pDONR P2R-P3 2A-TagBFP-NLS		this study
	pRPS5a::VENUS-2A-TagBFP	pB7m34GW	pDONR P4-P1R pRPS5a, pDONR221 VENUS-N7, pDONR P2R-P3 2A-TagBFP-NLS		this study
**marker lines**	pUBQ10::GID1a-mCherry	pB7m34GW	pDONR P4-P1R-pUBQ10 , pDONR221 GID1a , pDONR P2R-P3-mCherry		this study
	pCUC2::gai-1-VENUS	pB7m34GW	pDONR P4-P1R pCUC2 , pDONR221 gai-1 , pDONR P2R-P3 VENUS		this study
	pGID1b::2xmTQ2-GID1b	pGreen 0125	pDONR P4-P1R pGID1b , pDONR221 2xmTQ2 , pDONR P2R-P3 GID1b::tGID1b		this study
	pCUC2::LSSmOrange	pGreen	pDONR P4-P1R pCUC2 , pDONR221 LSSmOrange-N7 , pDONR P2R-P3 tNOS		this study
**Y2H (Fig.1b,c; Sup Table 1; Ext Data Fig.1b,c)**	pGBKT7 M5RGA	pGBKT7	pDONR207 M5RGA		this study
	pGBKT7 M5RGA^{m4}	pGBKT7	pDONR207 M5RGA^{m4}		this study
	pGBKT7 M5RGA^{m1}	pGBKT7	pDONR207 M5RGA^{m1}		this study
	pGBKT7 M5RGA^{m2}	pGBKT7	pDONR207 M5RGA^{m2}		this study
	pGBKT7 M5RGA^{m3}	pGBKT7	pDONR207 M5RGA^{m3}		this study
	pGADT7 GID1A	pGADT7	pDONR221 GID1A		this study
	pGADT7 GID1B	pGADT7	pDONR221 GID1B		this study
	pGADT7 GID1C	pGADT7	pDONR221 GID1C		this study
	pGADT7 TCP14	pGADT7	pDONR207 TCP14		this study
	pGADT7 JAZ1	pGADT7	pDONR207 JAZ1		this study
	pGADT7 IDD2	pGADT7	pDONR207 IDD2		this study
	pGADT7 BZR1	pGADT7			Salomé Prat
	pDEST32 M5RGA	pDEST32	pDONR207 M5RGA		this study
	pDEST32 M5RGA^{m2}	pDEST32	pDONR207 M5RGA^{m2}		this study
	Arabidopsis REGIA + REGULATOR library	pDEST22			ref. 71
**Co-IP (Fig.1e)**	p35S::IDD2-RFP	pB7RWG2	pDONR221 IDD2		this study
	p35S::TCP14-RFP	pB7WGR2	pDONR221 TCP14		this study
	p35S::RGA-GFP	pB7WGF2	pDONR221 RGA		this study
	p35S::RGA^{m2}-GFP	pB7WGF2	pDONR221 RGA^{m2}		this study
**Transactivation experiments (Fig.1f,g)**	pTCP::LUC			Miguel Blazquez	ref. 68
	pBRE::LUC			Miguel Blazquez	ref. 69
	p35S::3xHA-VP16-TCP14			Miguel Blazquez	ref. 68
	p35S::3xHA-VP16-BZR1			Miguel Blazquez	ref. 69
	p35S::RGA-GFP	pB7WGF2	pDONR221 RGA		this study
	p35S::RGA^{m2}-GFP	pB7WGF2	pDONR221 RGA^{m2}		this study

B. Lines generated in this paper.					
Lines				Number of independent transgenic lines	
N.	Genotype	Background	Plant selection agent	Obtained (T1)	Analyzed
1	*pRPS5a::RGA-VENUS-2A-TagBFP*	Col0	Basta	>20	3
2	*pRPS5a:: RGA ^{m4}-VENUS-2A-TagBFP*	Col0	Basta	>20	3
3	*pRPS5a:: RGA ^{m1}-VENUS-2A-TagBFP*	Col0	Basta	>20	3
4	*pRPS5a:: RGA ^{m3}-VENUS-2A-TagBFP*	Col0	Basta	>20	3
5	*pRPS5a::qmRGA*	Col0	Basta	>20	3
6	*pRPS5a::VENUS-2A-TagBFP*	Col0	Basta	10	3
7	*pUBQ10:: RGA-VENUS-2A-TagBFP*	Col0	Basta	>20	4
8	*pUBQ10:: RGA ^{m4}-VENUS-2A-TagBFP*	Col0	Basta	>20	3
9	*pUBQ10:: RGA ^{m1}-VENUS-2A-TagBFP*	Col0	Basta	>20	3
10	*pUBQ10::qmRGA*	Col0	Basta	>20	3
11	*pUBQ10:: RGA ^{m3}-VENUS-2A-TagBFP*	Col0	Basta	>20	3
12	*pCLV3::mCherry-NLS*	*pRPS5a::qmRGA*	Basta	obtained by crossing, 6 F3 homozygous analyzed	
13	*pUBQ10:: GID1a-mCherry*	Col0	Kan	>20	2
14		*pRPS5a::qmRGA*	Kan, Basta	>20	2
15		*pUBQ10::qmRGA*	Kan, Basta	>20	2
16	*pCUC2:: gai-1-VENUS*	Col0	Basta	>20	3
17	*pGID1b::2xmTQ2-GID1b*	Col0	Norfluorazone	25	2
18	*pCUC2::LSSmOrange*	Col0	Kan	12	3
19	*pRPS5a::qd17RGA*	Col0	Basta	40	4
20	*pRPS5a::qd17mRGA*	Col0	Basta	40	4
21	*pUBQ10::qd17RGA*	Col0	Basta	40	10
22	*pUBQ10::qd17mRGA*	Col0	Basta	40	4

C. Transgenic plant lines used in this work				
N.	Genotype	Background	Plant selection	Reference
1	*gai-t6 rga-t2 rgl1-1 rgl2-1*	*Ler*		Cheng et al., Development, 2004
2	*gai-t6 rga-t2 rgl1-1 rgl2-1 rgl3-4*	*Ler**		Koini et al., Current Biology, 2009. This mutant was constructed by crossing a gai-t6 rga-t2 rgl1-1 rgl2-1 homozygote with a rgl3-4homozygote (a T-DNA insertion from the publicly available SAIL collection [Columbia background]) that had been backcrossed six times successively onto the La-er background. A line homozygous for the mutant alleles at all five loci were selected from the F2 on the basis of resistance to paclobutrazol (a GA biosynthesis inhibitor) and further screened via PCR assay.
3	*pGID1a::GID1a-GUS*	Col0	Kan	Suzuki et al., Plant Journal, 2009.
4	*pGID1b::GID1b-GUS*	Col0	Kan	
5	*pGID1c::GID1c-GUS*	Col0	Kan	
6	*pGAI::GUS*	Col0		Gallego-Giraldo et al., Plant Journal, 2014.
7	*pRGA::GUS*	*Ler*		
8	*pRGL1::GUS*	Col0		
9	*rgl2-5 (a promoter trap GUS line)*	*Ler*	Kan	Lee et al., Genes and Development, 2002
10	*pRGA::GFP-RGA*	Col0	Kan	Silverstone. et al. Plant cell, 2001
11	*pRGL3::RGL3-GFP*	*Ler*	Basta	Wild et al., Plant cell, 2012
12	*pCLV3::mCherry-NLS*	Col0	Basta	Pfeiffer et al., eLife, 2016
13	*nlsGPS1*	Col0	Kan	Rizza et al., Nature Plants, 2017

Supplementary Table 2. Lists of all the plasmids (A) and transgenic lines generated (B) and the mutants and transgenic lines (C) we used and for this paper.

No	Name	Sequence (5'-3')	Usage
1	RGA.attB1.F	GGGGACAAGTTTGTACAAAAAAGCAGGCTTCATGAAGAGAGATCATCACCAATTCCA	Forward primer synthesizing RGA
2	RGA.attB2.R	GGGGACCACTTTGTACAAGAAAGCTGGGTTGTACGCCGCCGTCGAGAGTTTCCAAG	Reverse primer synthesizing RGA
3	RGAm1.2R	AGCCATAAGCGCGTGGACTAAAGCAGCAGCGTTCTCTTGCGAGTCAACC	Reverse primer synthesizing RGA^{m1}
4	RGAm1.3F	GGTTGACTCGCAAGAGAACGCTGCTGCTTTAGTCCACGCGCTTATGGCT	Forward primer synthesizing RGA^{m1}
5	RGAm2.2R	AATCAAACAGAGTCGAAGCAGCAGCTAACGATTCAGTAAAC	Reverse primer synthesizing RGA^{m2}
6	RGAm2.3F	GTTTACTGAATCGTTAGCTGCTGCTTCGACTCTGTTTGATT	Forward primer synthesizing RGA^{m2}
7	RGAm3.attB2.R	GGGGACCACTTTGTACAAGAAAGCTGGGTTGGTGGTAATGAGTGGACGAGTGTGCC	Reverse primer synthesizing RGA^{m3}
8	RGAm4.R	GGGGACCACTTTGTACAAGAAAGCTGGGTTGTACGCCGCCGTCGAGAGTTTCCAAGCGTCGGTGG	Reverse primer synthesizing RGA^{m4}
9	GID1a.attB1.F	GGGGACAAGTTTGTACAAAAAAGCAGGCTTCATGGCTGCGAGCGATGAAGTTAATCTT	Forward primer synthesizing GID1a
10	GID1a.attB2.R	GGGGACCACTTTGTACAAGAAAGCTGGGTTACATTCCGCGTTTACAAACGCCGAAA	Reverse primer synthesizing GID1a
11	M5RGA.attB1.F	GGGGACAAGTTTGTACAAAAAAGCAGGCTTCACGGCGGCGGGTGAGTCAACTCGTTC	Forward primer synthesizing M5RGA
12	VENUS.attB2.R	GGGGACCACTTTGTACAAGAAAGCTGGGTTGATAGATCTCTTGTACAGCTCGTC	Reverse primer synthesizing VENUS
13	GID1b.attB1.F	GGGGACAAGTTTGTACAAAAAAGCAGGCTTCATGGCTGGTGGTAACGAAGTCAACCTTAACGAA	Forward primer synthesizing GID1b
14	GID1b.attB2.R	GGGGACCACTTTGTACAAGAAAGCTGGGTTCTAAGGAGTAAGAAGCACAGGACTTGACTTGCTTT	Reverse primer synthesizing GID1b
15	GID1c.attB1.F	GGGGACAAGTTTGTACAAAAAAGCAGGCTTCATGGCTGGAAGTGAAGAAGTTAATCTTATTGAG	Forward primer synthesizing GID1c
16	GID1c.attB2.R	GGGGACCACTTTGTACAAGAAAGCTGGGTTTCATTGGCATTCTGCGTTTACAAATGCAGCTAT	Reverse primer synthesizing GID1c
17	pUBQ10.F	AAAGTCTGTATATATGACACAGAA	Forward primer synthesizing

			UBQ10 promoter
18	pHDG4.F	AAACGCTTTGTCGGTGATCTAAGAA	Forward primer synthesizing HDG4 promoter
19	pPDF1.F	ATAGCGGAATAGCTGGCAACTTCAA	Forward primer synthesizing PDF1 promoter
20	GID1a-CDS-F	TTAATATCCAACTTCAAAGTAGCC	Forward primer synthesizing probe of GID1a CDS (969 bp)
21	GID1a-CDS-R T7	TAATACGACTCACTATAGGG ACATTCCGCGTTTACAAACG	Reverse primer synthesizing probe of GID1a CDS
22	GID1a-3UTR-F	CACTGGGTTAGAGAAAGAAG	Forward primer synthesizing probe of GID1a 3’-UTR (557 bp)
23	GID1a-cDNA-R1914	TAATACGACTCACTATAGGG GGCTTTTTGAAACACATTATATC	Reverse primer synthesizing probe of GID1a 3’-UTR
24	GID1b-CDS-F40	AGAATTGTCCCACTCAACACATGGG	Forward primer synthesizing probe of GID1b CDS (1035 bp)
25	GID1B-antisenP1	TAATACGACTCACTATAGGG AGGAGTAAGAAGCACAGG	Reverse primer synthesizing probe of GID1b CDS
26	GID1B-senP2	CAACGACAATGTCTGGTCATG	Forward primer synthesizing probe of GID1b 3’-UTR (488 bp)
27	GID1B-antisenP2	TAATACGACTCACTATAGGG AATGCATGAACTAAAACAAGAAGG	Reverse primer synthesizing probe of GID1b 3’-UTR
28	GID1c-CDS-F	CTAATATCCAACTTTAAGCTAGC	Forward primer synthesizing probe of GID1c CDS (966 bp)
29	GID1c-CDS-R T7	TAATACGACTCACTATAGGG TTGGCATTCTGCGTTTAC	Reverse primer synthesizing probe of GID1c CDS
30	GID1c-3UTR-F	GAACACTCTTATCTCTCACTG	Forward primer synthesizing probe

			of GID1c 3'-UTR (545 bp)
31	GID1c-cDNA-R1819	TAATACGACTCACTATAGGG TAAGACTTATACATCAAATCTTTGTC	Reverse primer synthesizing probe of GID1c 3'-UTR
32	GAI_cDNA-F	TTGAGCTGTAGATGTTGCTGTTAG	Forward primer synthesizing probe of GAI cDNA (1061 bp)
33	GAI_cDNA-R T7	TAATACGACTCACTATAGGG AAGTGAGCGAACTTGAGA	Reverse primer synthesizing probe of GAI cDNA
34	GAI_3UTR-F	TAGATGGTGGCTCAATGAATTG	Forward primer synthesizing probe of GAI 3'-UTR (382 bp)
35	GAI_3UTR-R T7	TAATACGACTCACTATAGGG CTGGTCCACCTATAATACCATGC	Reverse primer synthesizing probe of GAI 3'-UTR
36	RGL1_CDS-F	ATGAAGAGAGAGCACAACCAC	Forward primer synthesizing probe of RGL1 cDNA (1530 bp)
37	RGL1_CDS-R T7	TAATACGACTCACTATAGGG CACACGATTGATTCGCC	Reverse primer synthesizing probe of RGL1 cDNA
38	RGL1_3UTR-F	ATGGGAAAAGTGAAAATGTGC	Forward primer synthesizing probe of RGL1 3'-UTR (310 bp)
39	RGL1_3UTR-R T7	TAATACGACTCACTATAGGG TCGTGTCATGAAACATTTATCC	Reverse primer synthesizing probe of RGL1 3'-UTR
40	RGL2_cDNA-F	GGAGTAAATTTCTTGGAGTTAGG	Forward primer synthesizing probe of RGL2 cDNA (1050 bp)
41	RGL2_cDNA-R T7	TAATACGACTCACTATAGGG TGAGCAAAATACGTAGCGAC	Reverse primer synthesizing probe of RGL2 cDNA
42	RGL2_3UTR-F	CGGTAGAGATGACTCGCC	Forward primer synthesizing probe of RGL2 3'-UTR (258 bp)

43	RGL2_3UTR-R T7	TAATACGACTCACTATAGGG CATACAGAATCGTTATCCTTCC	Reverse primer synthesizing probe of RGL2 3'-UTR
44	RGL3_cDNA-F	GACTATGACAGTCCATTGGACC	Forward primer synthesizing probe of RGL3 cDNA (1101 bp)
45	RGL3_cDNA-R T7	TAATACGACTCACTATAGGG ACGTCGTAACAGCTTCTAGAATC	Reverse primer synthesizing probe of RGL3 cDNA
46	RGL3_3UTR-F	TAGATACGTCGTCATAAAGAGG	Forward primer synthesizing probe of RGL3 3'-UTR (330 bp)
47	RGL3_3UTR-R T7	TAATACGACTCACTATAGGG CAAATTCATTCATGCGTCATTTTG	Reverse primer synthesizing probe of RGL3 3'-UTR
48	GID1b-attB4	GGGGACAACTTTGTATAGAAAAGTTGGGATTTGTTATGATCTTGTCGGG	Forward primer synthesizing GID1b promoter
49	GID1b-p3.9k-attB1r	GGGGACTGCTTTTTTGTACAAACTTGA AGTCTCCAAAACCCAGCAAAAAAG	Reverse primer synthesizing GID1b promoter
50	GID1b-attB2r-2	GGGGACAGCTTTCTTGTACAAAGTGGATATGGCTGGTGGTAACGAAG	Forward primer synthesizing GID1b cDNA plus terminator
51	GID1b-attB3	GGGGACAACTTTGTATAATAAAGTTGG GATTAACATAACCGCTCGTC	Reverse primer synthesizing GID1b cDNA plus terminator
52	RGAd17i2.F	GAGGTAACATGGACGAGGTTGCTTTGAAACTCGAAC	Internal forward primer synthesizing d17RGA
53	RGAd17i1.R	TTTCAAAGCAACCTCGTCCATGTTACCTCCACCGT	Internal reverse primer synthesizing d17RGA
54	JAZ1.attB1.F	GGGGACAAGTTTGTACAAAAAAGCAGGCTATATGTCGAGTTCTATGGAATGTTC	Forward primer synthesizing JAZ1
55	JAZ1.attB2.R	GGGGACCACTTTGTACAAGAAAGCTGGGTTTATTTCAGCTGCTAAACCGAG	Reverse primer synthesizing JAZ1
56	TCP14.attB1.F	GGGGACAAGTTTGTACAAAAAAGCAGGCTATATGCAAAAGCCAACATCAAG	Forward primer synthesizing TCP14
57	TCP14.attB2	GGGGACCACTTTGTACAAGAAAGCTGGGTTAT	Reverse primer

	.R	CTTGCTGATCCTC	synthesizing TCP14
58	IDD2.attB1.F	GGGGACAAGTTTGTACAAAAAAGCAGGCTATATGCCGGTAGATTTAGATAACTC	Forward primer synthesizing IDD2
59	IDD2.attB1.R	GGGGACCACTTTGTACAAGAAAGCTGGGTTTGATTTTCTTCTACTAATGTC	Reverse primer synthesizing IDD2

Supplementary Table 3. Oligos used in this study.

Supplementary Methods

Quantitative analysis of microscopy images

The images obtained from confocal microscopy are processed using a complex computational pipeline derived from the one introduced in [1]. It involves several steps of image analysis, computational geometry and data manipulation that allow going from raw 3D images to aligned quantified data points (Supplementary Fig. 17). In the following, we describe the methods used for these different steps, highlighting when new algorithms were developed with respect to the original methods from [1].

Automatic Cell Segmentation & Layer Estimation

Image Notations

We consider that a 3D image consists of an array I of size $K_x \times K_y \times K_z$ filled with values taken in an integer intensity interval $\mathcal{I} \subset \mathbb{N}$. The elements of this 3D array are called voxels. In the case of a 16-bit-encoded unsigned integer image, the intensity interval $\mathcal{I} = [\![0, 2^{16}[\![$. We denote:

$$I = \Big\{ I_{ijk} \in \mathcal{I} \;\mid\; (i,j,k) \in [\![0, K_x[\![\times [\![0, K_y[\![\times [\![0, K_z[\![\Big\}. \tag{1}$$

The voxels in the array grid can be projected into a physical space $\Omega \subset \mathbb{R}^3$ through a mapping function $\mathbf{x} : [\![0, K_x[\![\times [\![0, K_y[\![\times [\![0, K_z[\![\rightarrow \Omega$ that associates the array indices with a discrete, evenly spaced, 3D lattice $\Omega_I \subset \Omega$. The voxel size $v = (v_x, v_y, v_z) \in \mathbb{R}^3$ defines a spacing of the lattice that is potentially different on each dimension:

$$\Omega_I = \big\{ \mathbf{x}(i,j,k) = (i \cdot v_x, j \cdot v_y, k \cdot v_z) \;\mid\; (i,j,k) \in [\![0, K_x[\![\times [\![0, K_y[\![\times [\![0, K_z[\![\big\}. \tag{2}$$

This mapping allows to define the image as a function $I : \Omega_I \rightarrow \mathcal{I}$ that associates a 3D point $\mathbf{x} = (x, y, z)$ in the image definition space Ω_I with a fluorescence intensity value $I(\mathbf{x}) \in \mathcal{I}$, such that $\forall\, (i,j,k) \in [\![0, K_x[\![\times [\![0, K_y[\![\times [\![0, K_z[\![$,

$$I(\mathbf{x}(i,j,k)) = I_{ijk}. \quad (3)$$

In the case of a multichannel image, we denote I_S the image channel corresponding to the signal S. Cells are segmented for each meristem acquisition independently using the *Propidium Iodide* (PI) channel, which we denote I_{PI}.

Automatic Cell Segmentation

As described in [1], the image segmentation method we use is an auto-seeded 3D watershed algorithm derived from the MARS pipeline [2] applied to the I_{PI} image (Supplementary Fig. 18 a). The parameters are the following:

- The standard deviation of the Gaussian filter used for seed detection is $0.75\mu m$
- The h value used for the H-min transform is either 200 (for 16-bit images) or 2 (for 8-bit images)
- The standard deviation of the Gaussian filter used for watershed is $0.5\mu m$

In the end we obtain a segmented image I_{seg} that assigns an integer label to every voxel of the image grid Ω_I on which the image I_{PI} to segment is defined (Supplementary Fig. 18 b). The cells of the tissue are represented by independent connected regions of voxels (so that the same label can not be assigned to voxels that are not part of the same connected component of I_{seg}). The background corresponds to a specific label, that is systematically set to 1 to ensure consistency between images. Each cell labeled $c \in \mathcal{C} = [\![1, K_c]\!]$ is the then represented by a connected region Γ_c so that:

$$\Gamma_c = \{\mathbf{x} \in \Omega_I \mid I_{\text{seg}}(\mathbf{x}) = c\} \quad (4)$$

Tissue Surface Extraction

To determine cell layers and to estimate the curvature of L1 cells, the surface of the tissue is computed based on the I_{seg} image as a 3D triangle mesh $\mathcal{M} = \{\mathcal{V}, \mathcal{T}\}$, where:

- $\mathcal{V}$ is a set of vertices
- Each vertex $v \in \mathcal{V}$ is associated with a 3D position $M_v \in \mathbb{R}^3$

- $\mathcal{T}$ a set of triangles defined by triplets of indices $(v_1, v_2, v_3) \in \mathcal{V}^3$
- $\mathcal{T}$ is such that the resulting simplicial complex forms a 2-manifold [3] (Chapter 5.3: Topological Spaces, Chapter 6.3: Simplicial Complexes).

To obtain this triangle mesh, we use the binary mask I_{bin} of the segmented image I_{seg} that associates the values 0 to background voxels and 1 to tissue voxels:

$$\forall \mathbf{x} \in \Omega_I, I_{\text{bin}}(\mathbf{x}) = \begin{cases} 0 & \text{if} \quad I_{\text{seg}}(\mathbf{x}) = 1 \\ 1 & \text{otherwise.} \end{cases} \tag{5}$$

This binary image is meshed by applying a Marching Cubes algorithm [4] on a resampled version of the image, to ensure a cubic shape of the image voxels. This triangular mesh undergoes a phase of triangle decimation [5] and isotropic remeshing [6] to obtain a surface composed of roughly 50000 regular faces.

Topological Relationships between Cells

The notion of adjacency between cells in the segmented image I_{seg} is directly related to the notion of adjacency between the voxels of the underlying 3D matrix. There are 3 possible conceptions of voxel adjacency in 3D images, which can be summed up by:

- In *6-connectivity*, 2 voxels are adjacent if they share a common face, called a *surfel*
- In *18-connectivity*, 4 voxels are adjacent if they share a common edge, called a *linel*
- In *26-connectivity*, 8 voxels are adjacent if they share a common vertex, called a *pointel*

Note that each of these *topological elements* (of dimension 2, 1 and 0 respectively) can be associated with a 3D position, defined as the barycenter of the voxels that share it. For instance a *surfel* shared by voxels $\mathbf{x}$ and $\mathbf{x}'$ is represented by the 3D point $\frac{1}{2}(\mathbf{x} + \mathbf{x}')$.

We use this notion to define sets of *topological elements* that constitute the interface between cells. Depending on the number of cells considered (2, 3 or 4), those sets will consist respectively of *surfels*, *linels* or *pointels*. More precisely, we define:

- $\Gamma^2_{c,c'}$ as the set of 3D positions of *surfels* shared by voxels labeled c & c'
- $\Gamma^1_{c,c',c''}$ as the set of 3D positions of *linels* shared by voxels labeled c, c' & c''
- $\Gamma^0_{c,c',c'',c'''}$ as the set of 3D positions of *pointels* shared by voxels labeled c, c', c'' & c'''

With this framework, we consider that two cells are adjacent if their interface (composed of surfels) is larger than a minimal amount $n_{\min}$, so that $\forall c, c' \in \mathcal{C}$:

$$c \text{ is \textit{adjacent} to } c' \iff \left|\Gamma^2_{c,c'}\right| \geq n_{\min} \tag{6}$$

This adjacency definition is obviously symmetrical, and we denote $N(c)$ the set of cell labels c' such that c' is *adjacent* to c. Therefore $c' \in N(c) \iff c \in N(c')$. Practically, we used the value $n_{\min} = 8$ to determine cell adjacency.

Cell Layer Estimation

For the purpose of the analysis, we want to discriminate between the first layer of cells (L1) and the rest of the tissue. The most straightforward way to define L1 cells in segmented images is to consider the set $N(1)$ of cells that are adjacent to the background region. However, due to artifacts of the segmentation method, this criterion alone might cause cells from deeper layers to be wrongly identified as L1.

To overcome this issue, we use an additional criterion which is the proximity of the cell centers to the surface of the tissue, represented by the triangular surface mesh $\mathcal{M} = \{\mathcal{V}, \mathcal{T}\}$. The center C_c of the cell labeled c can be computed as:

$$C_c = \frac{1}{|\Gamma_c|} \sum_{\mathbf{x} \in \Gamma_c} \mathbf{x}. \tag{7}$$

For each cell labeled c, we identify the mesh vertex $v(c)$ that achieves the minimal distance to the cell center C_c: $\forall c \in \mathcal{C} \setminus \{1\}$,

$$v(c) = \underset{v \in \mathcal{V}}{\operatorname{argmin}} \left(\|C_c - M_v\| \right). \tag{8}$$

We define $\mathcal{L}_1$ as the subset of $\mathcal{C}$ formed by indices c of cells that are *closer* to the surface $\mathcal{M}$ than a distance threshold $d_{\max}$, while being adjacent to the background region in the segmented image. In other terms, $\forall c \in \mathcal{C} \setminus \{1\}$:

$$c \in \mathcal{L}_1 \iff \begin{cases} c \in N(1) \\ \|C_c - M_{v(c)}\| \leq d_{\max}. \end{cases} \tag{9}$$

In images of the shoot apex meristem, we use a maximal distance value of $d_{\max} = 7\mu m$, that roughly corresponds to the typical cell diameter in this tissue.

L1 Cell Curvature Estimation

By computing normal vectors on every vertex v of the surface mesh $\mathcal{M}$, it is possible to estimate the local curvature parameters of the surface [7]. We use this method compute the values of principal curvatures at the vertex v, denoted $\kappa^-(v)$ for the minimum curvature value and $\kappa^+(v)$ for the maximum curvature value. From these, we can derive the mean curvature value $\kappa(v) = \frac{1}{2}\left(\kappa^+(v) + \kappa^-(v)\right)$.
As a first approximation, we considered that the surface curvature of a L1 cell c could be measured by the curvature of the surface mesh $\mathcal{M}$ at its closest vertex $v(c)$. We estimate all L1 cell curvature parameters this way, denoted κ_c^-, κ_c^+ and κ_c for the minimum principal curvature, the maximal principal curvature and the mean curvature respectively, so that $\forall c \in \mathcal{L}_1$:

$$\kappa_c^- = \kappa^-(v(c)), \;\; \kappa_c^+ = \kappa^+(v(c)), \;\; \kappa_c = \kappa(v(c)) \tag{10}$$

Cell-level Nuclei Detection & Signal Quantification

To quantify accurately the information provided by the GA biosensor, it is necessary to look at fluorescence intensity not at the global cell scale but more finely, at the level of the nucleus where the fluorescent proteins are targeted. To do so, we look within each cell region $\Gamma_c \mid c \in \mathcal{C} \setminus \{1\}$ for the precise position of the nucleus center point.

Cell-level Nuclei Detection

To locate nuclei, we use the same 4D Gaussian scale-space transform [8] applied to the *pRPS5a::TagBFP* (BFP) channel, introduced in [1]. We obtain it by performing a convolution of the image I_{BFP} with a sequence of 3D isotropic Gaussian kernels of increasing standard deviations $\sigma \in \mathfrak{s}$. The scale interval $\mathfrak{s}$ is defined as a geometric sequence varying from $\sigma_{\min}$ to $\sigma_{\max}$.

This results in a 4D response image, denoted by extension $I_{\text{BFP}} : \Omega_I \times \mathfrak{s} \subset \mathbb{R}^4 \to \mathcal{I}$, which measures how the signal intensity locally forms a spherical blob of scale $\sigma \in \mathfrak{s}$.
But instead of detecting local maxima in this 4D image, we use the information from the segmented image to detect only one nucleus per cell (Supplementary Fig. 18 c). To do so, we retain for each cell labeled c, among all the voxels belonging to its region Γ_c, the position P_c of the voxel that achieves the highest response independently of scale, so that $\forall c \in \mathcal{C} \setminus \{1\}$:

$$P_c = \underset{\mathbf{x} \in \Gamma_c}{\text{argmax}} \left(\max_{\sigma \in \mathfrak{s}} \left(I_{\text{BFP}}(\mathbf{x}, \sigma) \right) \right). \tag{11}$$

We denote $\mathcal{P}$ the set of those nuclei points, identified by the integer label c of the cell region to which they belong, so that we can write:

$$\mathcal{P} = \{ P_c = (x_c, y_c, z_c) \mid c \in \mathcal{C} \setminus \{1\} \} . \tag{12}$$

The parameter values we used are the ones identified in the evaluation study performed in [1], namely $|\mathfrak{s}| = 3$, $\sigma_{\text{min}} = 0.8\mu m$ and $\sigma_{\text{max}} = 1.4\mu m$.

Nuclei Signal Quantification

We use the method of [1] to quantify the signal values at the level of the cell nuclei in each image channel separately. For the signal S, the signal value for the the cell labeled c is computed as the value of the image I_S filtered by a Gaussian kernel of radius $\sigma_{\mathcal{N}}$ at the voxel position P_c (Supplementary Fig. 18 d-e), so that $\forall c \in \mathcal{C} \setminus \{1\}$,

$$S_c = \left(I_S * G(\sigma_{\mathcal{N}}) \right) (P_c). \tag{13}$$

For example, the local level of expression of the $CLV3$ gene, imaged using *pCLV3::mCHERRY* in the channel I_{CLV3} would we quantified as $\text{CLV3}_c = \left(I_{\text{CLV3}} * G(\sigma_{\mathcal{N}}) \right) (P_c)$.
The standard deviation we used for the quantification of nuclei signals in SAM images is $\sigma_{\mathcal{N}} = 0.75\mu m$.

Nuclei Detection and Signal Quantification in Hypocotyl Images

In the specific case of hypocotyl images where no cell wall staining was performed, we rely on the nuclei detection method described in [1] to detect the nuclei in the I_{BFP} (Supplementary Fig. 18 g) and quantify both BFP and mRGAV signals (Supplementary Fig. 18 h). Given that the cell sizes are different from those of shoot apical meristems, where the best parameter values were identified, we adjusted the method parameters to perform best on those images. The parameter values we retained are $|\mathfrak{s}| = 4$, $\sigma_{\mathrm{min}} = 2.5\mu m$, $\sigma_{\mathrm{max}} = 5\mu m$ and $\sigma_{\mathcal{N}} = 1\mu m$.

Quantification of GA Signaling

In the case of the ratiometric GA sensor qmRGA, the level of mRGA-VENUS (abbreviated mRGAV from now on) measured as the intensity of the channel I_{mRGAV}, is normalized by dividing it by the intensity measured on the I_{BFP} channel that accounts notably for promoter activity. This way we compute a ratio of estimated signals for each nuclei point, so that $\forall c \in \mathcal{C} \setminus \{1\}$,

$$\mathrm{qmRGA}_c = \frac{\mathrm{mRGAV}_c}{\mathrm{BFP}_c}. \tag{14}$$

Finally, to represent the GA activity in the cell c, we estimate the quantity GA_c referred to as *GA Signaling*. Since mRGAV is degraded in presence of GA, the ratiometric value qmRGA_c is negatively related to the GA signaling activity. However, even if, by construction, the sensors for mRGAV and BFP are produced in a $1:1$ ratio, the intensities measured in I_{mRGAV} and I_{BFP} do not necessarily show a ratio of 1 (in the case no degradation occurs) due to the physical properties of the fluorescent proteins and the microscope settings used for their imaging. Therefore we considered the range of values taken by the qmRGA ratio across all observed samples, and retained the upper bound $\mathrm{qmRGA}_{\mathrm{max}}$ of these values, that we rounded to 3 for the sake of simplicity, as the value corresponding to the case where mRGAV is not degraded. In consequence, the positive quantity GA_c reflecting the GA signaling activity such that a value of 0 corresponds to no degradation (hence no GA signaling) is defined as $\forall c \in \mathcal{C} \setminus \{1\}$,

$$\mathrm{GA}_c = \mathrm{qmRGA}_{\max} - \mathrm{qmRGA}_c = 3 - \frac{\mathrm{mRGAV}_c}{\mathrm{BFP}_c}. \tag{15}$$

The formula is the same for both SAM images (Supplementary Fig. 18 f) and hypocotyl images (Supplementary Fig. 18 i). This quantity is the one displayed in Fig. 2g, Supplementary Fig. 3, Supplementary Fig. 4, Supplementary Fig. 5, Supplementary Fig. 6, Fig. 3d-g and Fig. 4f

Image Rigid Temporal Registration

The previous steps were performed individually on each frame of the time-lapse acquisitions. In our study, we focused on sequences of observations of the same individual over its development, consisting of K_t multichannel images $\{I(t_i) \mid i \in [\![0, K_t[\![\}$, indexed by their temporal position $t_i \in \mathbb{N}$ in hours relatively to the first time of acquisition $t_0 = 0h$. In the remaining, we will consistently index data computed from the i-th acquisition $I(t_i)$ by the temporal index. For instance $\mathcal{P}(t_i) = \{P_c \mid c \in \mathcal{C}(t_i) \setminus \{1\}\}$ denotes the set of nuclei points detected in $I(t_i)_{\mathrm{BFP}}$ using the cell regions defined by $I(t_i)_{\mathrm{Seg}}$.

Rigid Registration

Similarly to what was done in [1], we estimate 3D rigid transformations between consecutive time frames of a sequence using a block matching algorithm [9], in order to place the quantitative cell information of a given sequence in the same spatial reference frame. This estimation is performed on the *Propidium Iodide* channel $\{I(t_i)_{\mathrm{PI}} \mid i \in [\![0, K_t[\![\}$ of the consecutive images. This produces $K_t - 1$ isometry matrices in homogeneous coordinates $R_{t_i \leftarrow t_{i+1}}$ that can be inverted and/or multiplied to transform any frame of the sequence into the spatial reference frame of any other.

Registered Nuclei Points

We use the registration output to transform all the detected nuclei points into the coordinate system of the first frame of the sequence. By applying the resulting rigid transforms to the the nuclei points detected at time t_i, we obtain a new point cloud $\mathcal{P}(t_i)^0$, indexed by the

same set of integer cell labels $\mathcal{C}(t_i)$, such that $\forall c \in \mathcal{C}(t_i) \setminus \{1\}$,

$$P_c^0 = \left(\prod_{j=i-1}^{0} R_{t_j \leftarrow t_{j+1}} \right) P_c. \tag{16}$$

Cell Surfacic Growth Computation

Cellular growth is generally expressed in terms of *strain*, which represents a relative variation of length [10]. On a 3D surface, the strain is locally two-dimensional and can be described by two *strain values* $\gamma^+ > \gamma^-$ associated to two orthogonal *principal directions of growth*. Each value characterizes the change of length of a 2D element along the corresponding direction, as a percentage of its initial length.

Assuming that the epidermis of a given L1 cell can be approximated by a planar surface, we consider that *cell surfacic growth* can be described by two strain values and two principal directions of growth, which lie within the cell surface plane. The surfacic strain between two consecutive time points t_i and t_{i+1} is computed by comparing the surface of a cell c at time t_i with the combined surface of all its descendant cells at time t_{i+1}.

Manual L1 Cell Lineages

Cell lineages were manually generated by expertizing the segmented images $I(t_i)_{\mathrm{seg}}$ and $I(t_{i+1})_{\mathrm{seg}}$ obtained for two consecutive time points t_i and t_{i+1} (Supplementary Fig. 19 a-b). The rigid transformation was applied to the images to make the visual comparison of consecutive time points easier. The cell lineage itself $L(t_i, t_{i+1})$ consists of a set of couples of L1 cell labels (c, c') where:

- $c' \in \mathcal{L}_1(t_{i+1})$ is the label of a cell in the segmented image $I(t_{i+1})_{\mathrm{seg}}$.
- $c \in \mathcal{L}_1(t_i)$ is the label of the cell in the segmented image $I(t_i)_{\mathrm{seg}}$ that will give rise to the cell labelled c' at t_{i+1}.

We say that the cell c is the *mother cell* of the cell c', and conversely that the cell c' is a *daughter cell* of the cell c.

Cell Landmark Extraction

We choose to use the topological elements representing junctions between 2 or more cells in the segmented image as landmarks to compute the deformation of the tissue, as it is done in the literature [11]. However, to avoid relying only on the position of cell vertices, which can be very sensitive to segmentation artifacts, we use additional landmarks representing cell edges and cell interfaces, as proposed in [12] (Chapter 3.2.2: Quantifying cellular features).

We compute the landmark positions using the 3D positions of image topological elements (surfels, linels and pointels) at the interface between cells, and assign them a unique identifier consisting in a cell label tuple (Supplementary Fig. 19 d). More precisely to extract landmarks on a segmented image I_{seg}, we extract for each cell $c \in \mathcal{L}_1$:

- A landmark identified by (c) computed as the geometric median of $\Gamma^2_{1,c}$
- $\forall c' \in N(c) \cap \mathcal{L}_1$, a landmark identified by (c, c') computed as the geometric median of $\Gamma^1_{1,c,c'}$
- $\forall c', c'' \in N(c) \cap \mathcal{L}_1$, such that $c'' \in N(c')$, a landmark identified by (c, c', c'') computed as the geometric median of $\Gamma^0_{1,c,c',c''}$

Combining the segmented image $I(t_{i+1})_{\text{seg}}$ with the lineage information makes it possible to obtain a relabeled image where all the daughter cells of a cell c from $I(t_i)_{\text{seg}}$ carry the label c (Supplementary Fig. 19 b-c). We perform the landmark extraction process on both the *mother* segmented image $I(t_i)_{\text{seg}}$ (Supplementary Fig. 19 d) and the relabeled *daughter* image (Supplementary Fig. 19 e) to obtain two sets of landmark points that can then be paired using their cell label identifiers.

Surfacic Strain Estimation

For each cell $c \in \mathcal{L}_1(t_i)$ that has at least one daughter in $\mathcal{L}_1(t_{i+1})$, we are able to identify pairs of conserved cell landmark points between the mother and daughter segmented images. Each point is first projected on the 2D plane approximating the interface between the cell c and the background in its respective image. It is then centered by subtracting the projected position of the landmark identified by (c), representing the median point of the considered interface.

Based on the paired centered 2D positions (provided there are at least 4 of them), we compute a 2D transformation (rotation and scaling) by a linear regression without intercept, minimizing squared distance between pairs of landmarks [12, 11]. The singular value decomposition of the obtained 2D matrix allows to obtain the two strain values γ_c^+ and γ_c^- and their associated directions in the 2D cell plane. We reconstruct a 3D strain matrix in the reference frame of $I(t_{i+1})_{\text{seg}}$ by transforming the 2D matrix back into the projection base, assuming a strain value of 0 in the normal direction to the cell plane. This produces a "flat" tensor that represents the surfacic strain in 3D (Supplementary Fig. 19 f).

The surfacic strain estimation gives us several important growth features. We derive the *surfacic growth intensity* γ_c as the product of the strain values, so that $\forall c \in \mathcal{L}_1(t_i)$,

$$\gamma_c = \gamma_c^+ \cdot \gamma_c^- . \tag{17}$$

We also define the *surfacic growth anisotropy* a_c that measures how much the cell grows in a preferential direction. A growth anisotropy equal to 0 reflects the fact that growth is homogeneous in every direction, hence we define anisotropy as the norm of the deviatoric part of the strain tensor, normalized by the norm of its isotropic part. In the case of the 2D surfacic strain tensor, it is equivalent to define this measure as $\forall c \in \mathcal{L}_1(t_i)$,

$$a_c = \frac{\gamma_c^+ - \gamma_c^-}{\gamma_c^+ + \gamma_c^-}. \tag{18}$$

Curvature-based SAM Primordia Alignment

In order to pool cell data coming from several individuals SAMs, we perform a population alignment, similar to what was done in [1]. The general idea is to find a geometrical transformation that places organs with a similar state of development at the same location in the 3D space. This allows then to aggregate the data initially expressed in each individual's own image reference frame into a common reference frame where pointwise comparison is meaningful.

SAM Reference Frame

The target reference frame into which we aim to transform the data is the one introduced in [1]. It consists in a cylindrical coordinate system (r, θ, z) in which:

- the origin corresponds to the apex center in the central zone (CZ) of the meristematic dome
- the z-axis corresponds to the main rotational symmetry axis of the meristematic dome
- the *polar* axis ($\theta = 0$) corresponds to the direction of the last initiated primordium (P_0)
- the rotation orientation corresponds to the orientation (either clockwise or counter-clockwise) of the phyllotactic spiral.

Such a coordinate system can actually be mapped on the data by identifying the spatial positions of its key landmarks (CZ center $\mathbf{c} = (x_\mathbf{c}, y_\mathbf{c}, z_\mathbf{c}) \in \mathbb{R}^3$, unitary vertical axis $\mathbf{a} \in \mathbb{R}^3$, unitary P_0 radial vector $\mathbf{r} \in \mathbb{R}^3 \mid \mathbf{a} \perp \mathbf{r}$, and binary orientation $o \in \{-1, 1\}$). The computation of the alignment rigid geometric transformation from this set of landmarks is then direct.

2D Maps of L1 Signal

To detect the position of the landmarks in the cell-level data of a given individual SAM, we rely on a tool introduced in [1] that allows to infer a value of a signal S in any point of a 2D projection space, using the signal values of the subset $\mathcal{L}_1$ of first-layer cells. This computation relies on a 1D parametric sigmoid density function:

$$\begin{aligned} \eta \,:\, \mathbb{R} &\rightarrow [0,1] \\ r &\mapsto \frac{1}{2} - \frac{1}{2}\tanh\big(k \cdot (r - R)\big) \end{aligned} \tag{19}$$

for which we use the optimal parameter values evidenced in [1]: $R = 7.5\mu m$ and $k = 0.55\mu m^{-1}$. This allows to estimate a continuous signal map $\widehat{S}$ at any point $\mathbf{x} \in \mathbb{R}^2$ based on the projected point cloud of L1 cell points $\{P_c \in \mathbb{R}^2 \mid c \in \mathcal{L}_1\}$ and the associated signal values $\{S_c \mid c \in \mathcal{L}_1\}$ as: $\forall \mathbf{x} \in \mathbb{R}^2$,

$$\widehat{S}(\mathbf{x}) = \frac{1}{\sum\limits_{c \in \mathcal{L}_1} \eta\left(\|\mathbf{x} - P_c\|\right)} \sum_{c \in \mathcal{L}_1} \eta\left(\|\mathbf{x} - P_c\|\right) S_c. \tag{20}$$

We consider that the signal map $\widehat{S}(\mathbf{x})$ is defined only for points $\mathbf{x}$ where the total density $H(\mathbf{x}) = \sum_{c \in \mathcal{L}_1} \eta\left(\|\mathbf{x} - P_c\|\right)$ is greater than $\frac{1}{2}$.

SAM Center Detection

The *pCLV3::mCHERRY* image channel (Supplementary Fig. 20 a) provides a marker of the central zone (CZ) of the meristem. We use the same method as in [1] to estimate the 3D position of the CZ center $\mathbf{c} = (x_\mathbf{c}, y_\mathbf{c}, z_\mathbf{c})$, using the quantified signal values $\{\mathrm{CLV3}_n \mid n \in \mathcal{L}_1\}$.

The procedure relies on the computation of a 2D map $\widehat{\mathrm{CLV3}}$ based on the registered nuclei points $\mathcal{P}^0$ from all the time points of the sequence (Supplementary Fig. 20 b). This map allows to estimate a 2D position $(x_\mathbf{c}, y_\mathbf{c})$ of the CZ center, at which we compute the value of the altitude map $\widehat{z}$ to obtain the third coordinate of the center point $z_\mathbf{c}$.

Vertical Axis Optimization

Using the method from [1], we estimate the rotational symmetry axis $\mathbf{a}$ by looking for the direction that minimizes the dispersion of z coordinates of the CZ nuclei as a function of their radial distance.

Curvature-based Alignment

The two previous landmarks $\mathbf{c}$ and $\mathbf{a}$ allow to define a reference frame equipped with a cylindrical coordinate system (r, θ, z), in which the positioning of the direction $\mathbf{r}$ of the P_0 organ primordium comes down to the determination of an angular coordinate θ_0.

In [1], this was achieved by detecting the maximal point of the 2D map of Auxin signal in the peripheral zone (PZ) of the meristem. In our case however, the Auxin information is not available. Still, it is possible to indirectly use this information by using the SAM data aligned with the method from [1] as a geometrical reference for the alignment of new meristems.

We rely on the mean curvature information κ to represent accurately the geometrical information of the SAM surface in a 2D representation like the signal maps. Practically, we selected the $K_s = 21$ sequences of SAM acquisitions from [1] from which we use the aligned positions of L1 nuclei to compute K_s 2D aligned maps of mean curvature, noted $\left\{ \widehat{\kappa_j^*} \mid j \in [\![0, K_s[\![\right\}$ (Supplementary Fig. 20 c).

We use this set of reference maps to find optimal values for both the angular coordinate θ_0 of the P_0 primordium and the clockwise or counter-clockwise orientation o of the organ spiral by minimizing the error between the mean curvature map $\widehat{\kappa}$ obtained using nuclei points from all the time points of the considered sequence, rotated by the angle θ_0 and reflected if $o = -1$, and all the reference mean curvature maps $\widehat{\kappa_j^*}$.

The error between $\widehat{\kappa}$ and $\widehat{\kappa_j^*}$ is computed as the average of the pointwise absolute error over the intersection of the definition domains of both maps, restricted to the central and peripheral zones (PZ) of the SAM. We define the limits of the PZ by a radial distance threshold $r_{\text{PZ}} = 70\mu m$. This allows to delimit a 2D domain that actually depends of the considered rotation angle and orientation value:

$$\text{PZ}_j(\theta_0, o) = \left\{ (r, \theta) \in \mathbb{R}^2 \mid H(r, o \cdot (\theta - \theta_0)) > \frac{1}{2}, H_j^*(r, \theta) > \frac{1}{2}, r < r_{\text{PZ}} \right\}. \tag{21}$$

The optimal values θ_0^* and o^* (Supplementary Fig. 20 d) can then be written as:

$$(\theta_0^*, o^*) = \underset{\substack{\theta_0 \in [-\pi, \pi[\\ o \in \{-1, 1\}}}{\arg\min} \left(\frac{1}{K_s} \sum_{j=0}^{K_s - 1} \frac{1}{\iint_{\text{PZ}_j(\theta_0, o)} r\mathrm{d}r\mathrm{d}\theta} \iint_{\text{PZ}_j(\theta_0, o)} \left| \widehat{\kappa_j^*}(r, \theta) - \widehat{\kappa}(r, o \cdot (\theta - \theta_0)) \right| r\mathrm{d}r\mathrm{d}\theta \right). \tag{22}$$

The determination of θ_0 allows to position the radial vector $\mathbf{r}$, and the value of o achieves the definition of the cylindrical coordinate system (Supplementary Fig. 20 e)

Evaluation of curvature-based alignment

To evaluate this new alignment procedure, we applied it the 21 aligned SAM sequences we use as reference. We used a leave-one-out strategy, aligning the considered SAM by

minimizing the average curvature error with the 20 remaining series. In the ideal case, the optimal orientation o should be equal to 1 and the optimal P_0 angular coordinate value θ_0 should be equal to $0°$.

Our evaluation shows that the optimal orientation is always 1, and in the vast majority of the cases (72%) the optimal angle is such that $|\theta_0| \leq 6°$ (Supplementary Fig. 20 g). In the rest of cases, the optimal angle corresponds to one "golden" divergence angle of the organ phyllotactic spiral $\alpha^* \sim 137.5°$, as either $|\theta_0 + \alpha^*| \leq 6°$ (17%) or $|\theta_0 - \alpha^*| \leq 6°$ (11%). In other terms the alignment procedure either finds the right angle, or performs a shift of one primordium (aligning for instance P_1 on P_0. In any case, it manages to successfully superimpose organ primordia, but with a possible uncertainty on the equivalence of their developmental state. This temporal uncertainty is also explained by the fact that the reference SAMs themselves are not perfectly homogeneous in terms of developmental state. The fact that most alignment angles are correct, and that both negative and positive primordium shifts exist in the dataset confirms that the reference SAMs are "centered" on what can be seen as a sound characterization of the P_0 stage, and validates the approach of using them as a geometrical reference.

Aligned L1 nuclei points

In the end, the determination of the SAM landmarks $\mathbf{c}$, $\mathbf{a}$, $\mathbf{r}$ and o allows to transform the sequence registered points $\mathcal{P}^0$ into the common 3D reference frame in which we will be able to compare different individuals locally, as described in [1].

We note $\mathcal{P}^* = \{P_c^* \mid c \in \mathcal{L}_1\}$ the positions of first-layer nuclei points in this common reference frame. The 2D maps computed using these aligned positions (Supplementary Fig. 20 f) make it possible to perform pointwise inter-individual comparisons, as the data points that are superimposed at a given location correspond to tissue areas in a similar state of development. Therefore, it opens the way to population statistics.

These nuclei positions are the ones displayed in Fig. 4g, and were used to compute the average GA signaling map displayed in Fig. 4f and the average surfacic growth intensity and surfacic growth anisotropy maps displayed in Fig. 5a-b.

Synthesis and characterization of GA-fluorescein (GA-Fl)

General chemistry methods and instrumentation

GA_3, GA_4 and GA_7 were purchased from Duchefa Biochemie. All other chemicals were purchased from Merck or Combi-Blocks and were used as received unless otherwise stated. Anhydrous solvents and reagents were obtained as SureSeal bottles from Merck. Thin-layerchromatography and flash chromatography were performed using Merck KGaA pre-coated silica gel 60 F-254 plates and Silicycle silica gel 40-63 (230-400 mesh), respectively. UV absorbance spectra were recorded on Agilent Cary 60 UV-Vis Spectrophotometer. Fluorescence spectra were recorded on Fluorolog 2 (Spex) fluorimeter. Low resolution ESI mass spectrometry was performed on LC/MS Acquity QDa detector coupled with Waters HPLC. High resolution ESI mass spectrometry was performed on a Waters SYNAPT system. ^{1}H and ^{13}C NMR spectra were collected in DMSO-d6 (Cambridge Isotope Laboratories, Cambridge, MA) at 25°C using a Bruker Advance III spectrometer at 400 MHz and 100 MHz respectively at the Department of Chemistry NMR Facility at Tel-Aviv University. All chemical shifts are reported in the standard δ notation of parts per million using either TMS or residual solvent peak as an internal reference. Abbreviations: MeCN: acetonitrile, DMF: dimethylformamide, HATU: Hexafluorophosphate Azabenzotriazole Tetramethyl Uronium, TFA: Trifluoroacetic acid, DIPEA: *N*,*N*-Diisopropylethylamine.

HPLC-MS Analysis conditions

HPLC-MS analysis was performed on Waters HPLC with XBridge C18 column (100 X 3 mm, 5 μm), starting with 2 min of solvent A (water), followed by a water-acetonitrile gradient from 0% to 100% solvent B (acetonitrile) in 15 minutes then 1 minute at 100% solvent B and ending with 2 min 100% solvent A at flow rate of 1 mL/min (solvent A = water, solvent B = acetonitrile, both contain 0.1% TFA as an additive). Mass spectrometry was performed on LC/MS Acquity QDa detector coupled with Waters HPLC.

Preparative HPLC purification conditions

Preparative HPLC was performed on Waters 2545 HPLC with XBridge C18 column (100 X 19 mm, 5 μm) starting with 2 min of solvent A (water), followed by a water-acetonitrile gradient from 0% to 80% solvent B (acetonitrile) in 20 minutes, continuing with 3 min gradient of 80% to 100% solvent B then 3 minutes at 100% solvent B and ending with 2 min 100% solvent A at flow rate of 15 mL/min (solvent A = water, solvent B = acetonitrile, both contain 0.1% TFA as an additive).

Synthesis of GA-Fluorescein (GA-Fl) derivatives

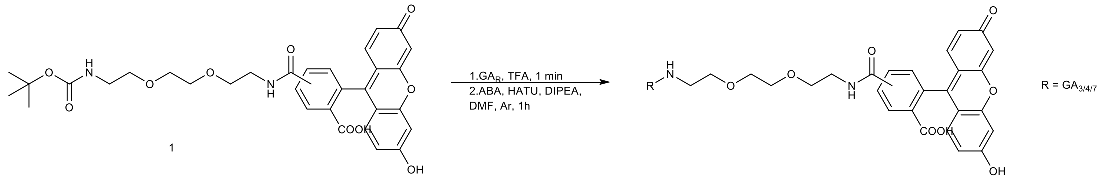

Briefly, compound **1** (10 mg, 1 eq) was dissolved in 0.5 mL TFA and stirred for 1 minute. TFA was immediately removed under reduced pressure. The residue was dissolved in dry DMF under argon atmosphere and DIPEA (3.3 μL, 1.1 eq) was added. In a separate 1.5 mL eppendorf, the appropriate GA (3, 4 or 7, 1.1 eq) was dissolved in dry DMF, then DIPEA (7.6 μL, 1.1 eq) and HATU (7.2 mg, 1.1 eq) were added. The mixture was vortexed for 2 minute and was then added to the TFA-treated compound **1**. The reaction was stirred at room temperature for 1 hour and the solvent was removed under reduced pressure. The residue was dissolved in 2 mL acetonitrile and the desired product was purified using preparative HPLC (see preparative HPLC purification conditions above) [13].

GA_3-Fl (Supplementary Figure 21 and 22): Preparative HPLC retention time: 13.43 min. Obtained 10 mg yellow solid (12 μmol, yield 68%). ^{1}H NMR (400 MHz, DMSO) δ 10.16 (s, 1H), 8.89 (d, J = 5.5 Hz, 1H), 8.74 (s, 1H), 8.44 (d, J = 1.6 Hz, 1H), 8.22 (dd, J = 8.1, 1.6 Hz, 1H), 8.17 – 8.01 (m, 2H), 7.35 (d, J = 8.0 Hz, 1H), 6.67 (d, J = 2.3 Hz, 1H), 6.59 – 6.49 (m, 3H), 6.30 (dd, J = 9.3, 3.2 Hz, 1H), 5.76 (td, J = 9.4, 3.6 Hz, 1H), 5.56 (d, J = 6.6 Hz, 1H), 5.48 (dd, J = 6.7, 2.0 Hz, 1H), 5.10 (s, 1H), 5.03 (s, 1H),

4.82 (s, 1H), 4.76 (d, *J* = 4.7 Hz, 1H), 3.83 (ddd, *J* = 13.2, 7.0, 3.9 Hz, 2H), 3.57 – 3.39 (m, 9H), 3.11 – 3.02 (m, 2H), 2.55 (s, 1H), 2.14 (d, *J* = 8.0 Hz, 1H), 2.05 (s, 1H), 1.90 – 1.51 (m, 9H), 1.21 (s, 1H), 1.07 – 1.00 (m, 3H). ^{13}C NMR (101 MHz, DMSO) δ 171.29, 160.09, 152.29, 133.76, 131.98, 129.58, 124.69, 113.14, 106.63, 105.94, 102.74, 91.36, 77.24, 77.05, 69.99, 69.53, 53.57, 50.91, 50.19, 49.86, 44.77, 17.00, 14.95, 14.67.; LC/MS: Retention time 9.15 min, 835.33 $[M+H]^+$. HR-MS(ESI) calcd. for formula $C_{46}H_{47}N_2O_{13}$ $[M+H]^+$: 835.3076; found: 835.3078.

GA_4-Fl (Supplementary Figure 23 and 24): Preparative HPLC retention time: 16.23 min. Obtained 10 mg yellow solid (12 μmol, yield 70%). ^{1}H NMR (400 MHz, DMSO) δ 9.98 (s, 1H), 8.71 (t, *J* = 5.5 Hz, 1H), 8.29 (d, *J* = 1.6 Hz, 1H), 8.07 (dd, *J* = 8.0, 1.6 Hz, 1H), 7.93 – 7.79 (m, 1H), 7.20 (d, *J* = 8.1 Hz, 1H), 6.52 (t, *J* = 2.6 Hz, 1H), 6.39 (dtq, *J* = 8.7, 4.5, 2.0 Hz, 2H), 4.70 (d, *J* = 8.3 Hz, 1H), 4.60 (d, *J* = 7.2 Hz, 1H), 3.37 – 3.26 (m, 7H), 3.03 – 2.87 (m, 7H), 2.36 (d, *J* = 1.9 Hz, 1H), 2.31 (d, *J* = 1.9 Hz, 1H), 1.84 (dd, *J* = 12.5, 5.4 Hz, 3H), 1.72 – 1.62 (m, 2H), 1.59 – 1.48 (m, 3H), 1.41 – 1.25 (m, 6H), 1.12 – 1.04 (m, 7H), 0.81 (t, *J* = 6.2 Hz, 2H), 0.68 (t, *J* = 6.5 Hz, 1H).^{13}C NMR (101 MHz, DMSO) δ 160.06, 157.78, 152.29, 129.59, 113.13, 109.56, 106.91, 102.73, 94.30, 69.98, 69.52, 69.24, 69.15, 54.76, 53.37, 52.56, 51.18, 50.99, 44.84, 37.35, 33.79, 31.54, 29.46, 27.29, 24.91, 22.53, 16.12, 14.87.; LC/MS: Retention time 10.23 min, 821.27 $[M+H]^+$. HR-MS(ESI) calcd. for formula $C_{46}H_{47}N_2O_{12}$ $[M-H]^+$: 819.3129; found: 819.3123.

GA_7-Fl (Supplementary Figure 25 and 26): Preparative HPLC retention time: 16.44 min. Obtained 9 mg yellow solid (11 μmol, yield 63%). ^{1}H NMR (400 MHz, DMSO) δ 10.12 (s, 1H), 8.72 (t, *J* = 5.5 Hz, 1H), 8.15 (dd, *J* = 8.1, 1.4 Hz, 1H), 8.06 (d, *J* = 8.1 Hz, 1H), 8.01 (d, *J* = 6.0 Hz, 1H), 7.66 (d, *J* = 1.1 Hz, 1H), 6.67 (t, *J* = 2.6 Hz, 1H), 6.60 – 6.50 (m, 3H), 6.31 (d, *J* = 9.3 Hz, 1H), 5.75 (dd, *J* = 9.3, 3.6 Hz, 1H), 4.86 (s, 1H), 4.75 (s, 1H), 3.82 (s, 1H), 3.56 – 3.38 (m, 9H), 3.21 – 2.99 (Im, 10H), 2.56 – 2.52 (m, 1H), 2.45 (s, 1H), 1.98 (s, 2H), 1.90 – 1.73 (m, 3H), 1.51 (tt, *J* = 17.4, 9.1 Hz, 4H), 1.31 – 1.19 (m, 2H), 1.03 (d, *J* = 6.5 Hz, 2H), 0.95 (d, *J* = 6.5 Hz, 1H).^{13}C NMR (101

MHz, DMSO) δ 179.60, 178.98, 171.19, 171.17, 168.49, 165.06, 160.09, 158.49, 158.18, 157.74, 157.70, 153.17, 152.30, 141.02, 136.63, 135.11, 133.67, 132.17, 129.85, 129.66, 128.66, 125.31, 122.74, 113.20, 109.60, 107.09, 102.72, 91.63, 83.73, 69.98, 69.88, 69.52, 69.45, 69.23, 69.09, 68.99, 53.56, 52.11, 51.95, 51.52, 44.99, 38.94, 36.93, 31.49, 15.87, 14.71.; LC/MS: Retention time 10.30 min, 819.30 $[M+H]^+$. HR-MS(ESI) calcd. for formula $C_{46}H_{45}N_2O_{12}$ $[M-H]^+$: 817.2973; found: 817.2961.

Supplementary References

[1] Galvan-Ampudia, C. *et al*. Temporal integration of auxin information for the regulation of patterning. *eLife* **9,** e55832 (2020).

[2] Fernandez, R. *et al*. Imaging plant growth in 4D: robust tissue reconstruction and lineaging at cell resolution. *Nature Methods* **7**, 547–553 (2010).

[3] Agoston, M. Computer graphics and geometric modeling, volume 1. Springer, (2005).

[4] Lorensen, W and Cline, H. Marching cubes: a high resolution 3d surface construction algorithm. *In Proceedings of the 14th Annual Conference on Computer Graphics and Interactive Techniques*, SIGGRAPH '87, pages 163–169 (1987).

[5] Garland, M. and Heckbert, P. Surface simplification using quadric error metrics. *In Proceedings of the 24th annual conference on Computer graphics and interactive techniques*, SIGGRAPH '97, pages 209–216 (1997).

[6] Botsch, M. and Kobbelt, L. A remeshing approach to multiresolution modeling. *In Proceedings of the Symposium on Geometry Processing*, pages 185–192 (2004).

[7] Theisel, H. Normal based estimation of the curvature tensor for triangular meshes. *In Pacific Conference on Computer Graphics and Applications*, pages 288–297, 2004.

[8] Lindeberg, T. Scale-space theory: A basic tool for analyzing structures at different scales. *J. Appl. Stat.* **21**, 225–270 (1994).

[9] Ourselin, S. *et al*. Block Matching : A general framework to improve robustness of rigid registration of medical images. *Medical Image Computing and Computer-Assited Intervention* **1935**, 557–566 (2000).

[10] Dumais, J. and Kwiatkowska, D. Analysis of surface growth in shoot apices. *Plant J.* **31**, 229–241 (2002).

[11] Barbier de Reuille, P. *et al*. Morphographx: A platform for quantifying morphogenesis in 4D. *eLife* **4**, e05864 (2015).

[12] Legrand, J. Toward a multi-scale understanding of flower development - from auxin networks to dynamic cellular patterns. *Theses, Ecole normale supérieure de lyon - ENS LYON*, November 2014.

[13] Shani, E. *et al.* Gibberellins accumulate in the elongating endodermal cells of *Arabidopsis* root. *Proc. Natl. Acad. Sci.* **110**, 4834–4839 (2013).
