# Supplementary material for: A quantitative gibberellin signaling biosensor reveals a role for gibberellins in internode specification at the shoot apical meristem
Source: Nat Commun. 2024 May 8;15:3895. doi: 10.1038/s41467-024-48116-4 (PMC11079023; doi:10.1038/s41467-024-48116-4)
Supplement: Supplementary file 3 — Reporting Summary [file 41467_2024_48116_MOESM3_ESM.pdf]

nature portfolio

Corresponding author(s): Teva Vernoux, Patrick Achard

Last updated by author(s): Apr 8, 2024

Reporting Summary

Nature Portfolio wishes to improve the reproducibility of the work that we publish. This form provides structure for consistency and transparency in reporting. For further information on Nature Portfolio policies, see our Editorial Policies and the Editorial Policy Checklist.

Statistics

For all statistical analyses, confirm that the following items are present in the figure legend, table legend, main text, or Methods section.

n/a	Confirmed	
☐	☒	The exact sample size (*n*) for each experimental group/condition, given as a discrete number and unit of measurement
☐	☒	A statement on whether measurements were taken from distinct samples or whether the same sample was measured repeatedly
☐	☒	The statistical test(s) used AND whether they are one- or two-sided<br>*Only common tests should be described solely by name; describe more complex techniques in the Methods section.*
☒	☐	A description of all covariates tested
☐	☒	A description of any assumptions or corrections, such as tests of normality and adjustment for multiple comparisons
☐	☒	A full description of the statistical parameters including central tendency (e.g. means) or other basic estimates (e.g. regression coefficient) AND variation (e.g. standard deviation) or associated estimates of uncertainty (e.g. confidence intervals)
☐	☒	For null hypothesis testing, the test statistic (e.g. *F*, *t*, *r*) with confidence intervals, effect sizes, degrees of freedom and *P* value noted<br>*Give P values as exact values whenever suitable.*
☒	☐	For Bayesian analysis, information on the choice of priors and Markov chain Monte Carlo settings
☒	☐	For hierarchical and complex designs, identification of the appropriate level for tests and full reporting of outcomes
☒	☐	Estimates of effect sizes (e.g. Cohen's *d*, Pearson's *r*), indicating how they were calculated

Our web collection on statistics for biologists contains articles on many of the points above.

Software and code

Policy information about availability of computer code

Data collection	No software was used for data collection
Data analysis	Fiji (fiji.sc), Zeiss Zen 2, VTK library, MorphoGraphX for image analysis ; Python libraries for image analysis: timagetk, cellcomplex, tissue_nukem_3d and sam_atlas (https://gitlab.inria.fr/mosaic/), sam_spaghetti ((https://gitlab.inria.fr/mosaic/publications/sam_spaghetti), and qrga_nuclei_quantification (https://gitlab.inria.fr/gcerutti/); R, Excel and http://www.physics.csbsju.edu/stats/KS-test.n.plot_form.html for statistical analysis

For manuscripts utilizing custom algorithms or software that are central to the research but not yet described in published literature, software must be made available to editors and reviewers. We strongly encourage code deposition in a community repository (e.g. GitHub). See the Nature Portfolio guidelines for submitting code & software for further information.

Data

Policy information about availability of data

All manuscripts must include a data availability statement. This statement should provide the following information, where applicable:

- Accession codes, unique identifiers, or web links for publicly available datasets
- A description of any restrictions on data availability
- For clinical datasets or third party data, please ensure that the statement adheres to our policy

All data generated and analyzed during this study are either included in this published article (and its supplementary information files) or available for download from Zenodo (DOI: 10.5281/zenodo.10934411). Source data are provided with this paper.

Research involving human participants, their data, or biological material

Policy information about studies with human participants or human data. See also policy information about sex, gender (identity/presentation), and sexual orientation and race, ethnicity and racism.

Reporting on sex and gender	N/A
Reporting on race, ethnicity, or other socially relevant groupings	N/A
Population characteristics	N/A
Recruitment	N/A
Ethics oversight	N/A

Note that full information on the approval of the study protocol must also be provided in the manuscript.

Field-specific reporting

Please select the one below that is the best fit for your research. If you are not sure, read the appropriate sections before making your selection.

☒ Life sciences ☐ Behavioural & social sciences ☐ Ecological, evolutionary & environmental sciences

For a reference copy of the document with all sections, see nature.com/documents/nr-reporting-summary-flat.pdf

Life sciences study design

All studies must disclose on these points even when the disclosure is negative.

Sample size	Sample size were chosen accordingly to the minimal sample size requirements for statistical tests
Data exclusions	No data were excluded
Replication	All experiments were reproduced
Randomization	No randomization was used. Each statistical test uses simple experimental design
Blinding	Blinding was not possible since we know the nature of all samples for each experiments.

Reporting for specific materials, systems and methods

We require information from authors about some types of materials, experimental systems and methods used in many studies. Here, indicate whether each material, system or method listed is relevant to your study. If you are not sure if a list item applies to your research, read the appropriate section before selecting a response.

Materials & experimental systems

n/a	Involved in the study	
☐	☒	Antibodies
☒	☐	Eukaryotic cell lines
☒	☐	Palaeontology and archaeology
☒	☐	Animals and other organisms
☒	☐	Clinical data
☒	☐	Dual use research of concern
☐	☒	Plants

Methods

n/a	Involved in the study	
☒	☐	ChIP-seq
☒	☐	Flow cytometry
☒	☐	MRI-based neuroimaging

Antibodies

Antibodies used	Anti-digoxigenin-AP, Fab fragments, Roche, Catalog number: 11 093 274 910 ; anti-GFP antibody conjugated with paramagnetic beads (Miltenyi Biotec, Catalog number 130-091-125); anti-GFP (JL8; Clontech, Catalog number 632380); anti-RFP (6G6 α-Red, Chromotek, Catalog number 51020014AB); anti-RGA (Agrisera, Catalog numberAS111630); anti-HA (Sigma, Catalog number H9658, clone HA-7); anti-actin (Agrisera, Catalog number AS132640); peroxidase-conjugated goat anti-rabbit or mouse IgG (Invitrogen, Catalog number G21040 and G21234, respectively)
Validation	Validation of the antibodies is available from the website of the companies (https://www.sigmaaldrich.com/catalog/product/roche/11093274910?lang=en&region=GB; https://www.miltenyibiotec.com/FR-en/products/umacs-and-multimacs-gfp-isolation-kits.html#130-091-125;https://www.takarabio.com/documents/Certificate%20of%20Analysis/632380/632380-632381-070313.pdf;https://www.ptglab.com/products/RFP-antibody-6G6.htm; https://www.agrisera.com/en/artiklar/rga-della-protein-rga.html; https://www.sigmaaldrich.com/US/en/product/sigma/h9658; https://www.agrisera.com/en/artiklar/act-actin.html); see also e.g. Hejatko J et al. Nature Protocols 2006 1:1939-1946, Rozier F et al 2014 Nature Protocols 9: 2464-2475, Davière J-M et al. Current Biology 24:1923-1928

Dual use research of concern

Policy information about dual use research of concern

Hazards

Could the accidental, deliberate or reckless misuse of agents or technologies generated in the work, or the application of information presented in the manuscript, pose a threat to:

No	Yes	
☒	☐	Public health
☒	☐	National security
☒	☐	Crops and/or livestock
☒	☐	Ecosystems
☒	☐	Any other significant area

Experiments of concern

Does the work involve any of these experiments of concern:

No	Yes	
☒	☐	Demonstrate how to render a vaccine ineffective
☒	☐	Confer resistance to therapeutically useful antibiotics or antiviral agents
☒	☐	Enhance the virulence of a pathogen or render a nonpathogen virulent
☒	☐	Increase transmissibility of a pathogen
☒	☐	Alter the host range of a pathogen
☒	☐	Enable evasion of diagnostic/detection modalities
☒	☐	Enable the weaponization of a biological agent or toxin
☒	☐	Any other potentially harmful combination of experiments and agents

Plants

Seed stocks	Supplementary Table 2 lists all the published lines used in this study together with the corresponding publications.
Novel plant genotypes	Novel plant genotypes were generated by transgenic approaches using floral dipping. Supplementary Table 2 lists all the transgenic plants generated in the study. The number of independent lines analyzed for each novel genotype is indicated in the manuscript in the Supplementary Table 2. Experiments were performed using homozygous plants from the T3 generation.
Authentication	Plant were characterized using antibiotic resistance analysis, phenotype analysis and comparison to published phenotyping data for the mutants, as well as using fluorescence expression analysis for the lines expressing a fluroescent protein. For the lines generated in this study, potential secondary effects from a second site T-DNA insertion was examined first by selecting only plants with a 3:1 segregation of the antibiotic resistance, and then by verifying consistency of phenotypes or expression of fluorescent proteins across independent lines.
